# Gap Junctions in A8 Amacrine Cells Are Made of Connexin36 but Are Differently Regulated Than Gap Junctions in AII Amacrine Cells

**DOI:** 10.3389/fnmol.2019.00099

**Published:** 2019-04-23

**Authors:** Shubhash C. Yadav, Stephan Tetenborg, Karin Dedek

**Affiliations:** ^1^Animal Navigation/Neurosensorics, Institute for Biology and Environmental Sciences, University of Oldenburg, Oldenburg, Germany; ^2^Research Center Neurosensory Science, University of Oldenburg, Oldenburg, Germany

**Keywords:** amacrine cell, bipolar cell, gap junction, electrical synapse, connexin36, retina, dopamine

## Abstract

In the mammalian retina, amacrine cells represent the most diverse cell class and are involved in the spatio-temporal processing of visual signals in the inner plexiform layer. They are connected to bipolar, other amacrine and ganglion cells, forming complex networks via electrical and chemical synapses. The small-field A8 amacrine cell was shown to receive non-selective glutamatergic input from OFF and ON cone bipolar cells at its bistratified dendrites in sublamina 1 and 4 of the inner plexiform layer. Interestingly, it was also shown to form electrical synapses with ON cone bipolar cells, thus resembling the rod pathway-specific AII amacrine cell. In contrast to the AII cell, however, the electrical synapses of A8 cells are poorly understood. Therefore, we made use of the Ier5-GFP mouse line, in which A8 cells are labeled by GFP, to study the gap junction composition and frequency in A8 cells. We found that A8 cells form <20 gap junctions per cell and these gap junctions consist of connexin36. Connexin36 is present at both OFF and ON dendrites of A8 cells, preferentially connecting A8 cells to type 1 OFF and type 6 and 7 ON bipolar cells and presumably other amacrine cells. Additionally, we show that the OFF dendrites of A8 cells co-stratify with the processes of dopaminergic amacrine cells from which they may receive GABAergic input via GABA_A_ receptor subunit α3. As we found A8 cells to express dopamine receptor D_1_ (but not D_2_), we also tested whether A8 cell coupling is modulated by D_1_ receptor agonists and antagonists as was shown for the coupling of AII cells. However, this was not the case. In summary, our data suggests that A8 coupling is differently regulated than AII cells and may even be independent of ambient light levels and serve signal facilitation rather than providing a separate neuronal pathway.

## Introduction

In the inner plexiform layer (IPL) of the mouse retina, almost 100 different neurons (bipolar, amacrine and ganglion cells) are interconnected in neuronal pathways. Amacrine cells probably represent the most numerous class comprising more than 40 different cell types (Helmstaedter et al., 2013), each with unique properties, such as dendritic pattern, synaptic partners, and consequently physiological function. Amacrine cells are commonly divided according to their dendritic tree size into small-field and wide-field amacrine cells (Kolb et al., 1981). In the mammalian retina, this subdivision is also reflected in the neurotransmitter used by the cells: small-field cells are glycinergic (Menger et al., 1998), whereas wide-field cells are GABAergic (Chen et al., 2011). In the following, we will focus on small-field amacrine cells whose dendrites typically extend <200 μm and span several sublaminae in the IPL, thereby often crossing the OFF-ON boundary between sublamina 2 and 3 (Werblin, 2011).

Among the small-field amacrine cells, the bistratified AII amacrine cell is the best studied, revealing a highly complex synaptic network (Tsukamoto and Omi, 2013, 2017; Marc et al., 2014). This synaptic network comprises not only glycinergic synapses to OFF bipolar cells and—to a smaller degree—OFF ganglion cells, but also two distinct gap junctional networks, one among AII cells (homocellular coupling) and one between AII and ON cone bipolar cells (heterocellular coupling). AII amacrine cells and their gap junction circuitry have been extensively studied, highlighting the molecular composition (Feigenspan et al., 2001; Maxeiner et al., 2005; Meyer et al., 2016), assembly (Meyer et al., 2014), and remarkable plasticity of AII gap junctions (Mills and Massey, 1995; Bloomfield and Völgyi, 2004; Kothmann et al., 2009). Moreover, AII cells are essential elements of the primary rod pathway (Güldenagel et al., 2001; Deans et al., 2002) in that they collect scotopic signals from rod bipolar cells and transmit them via gap junctions to ON cone bipolar cells.

**Figure 1:**
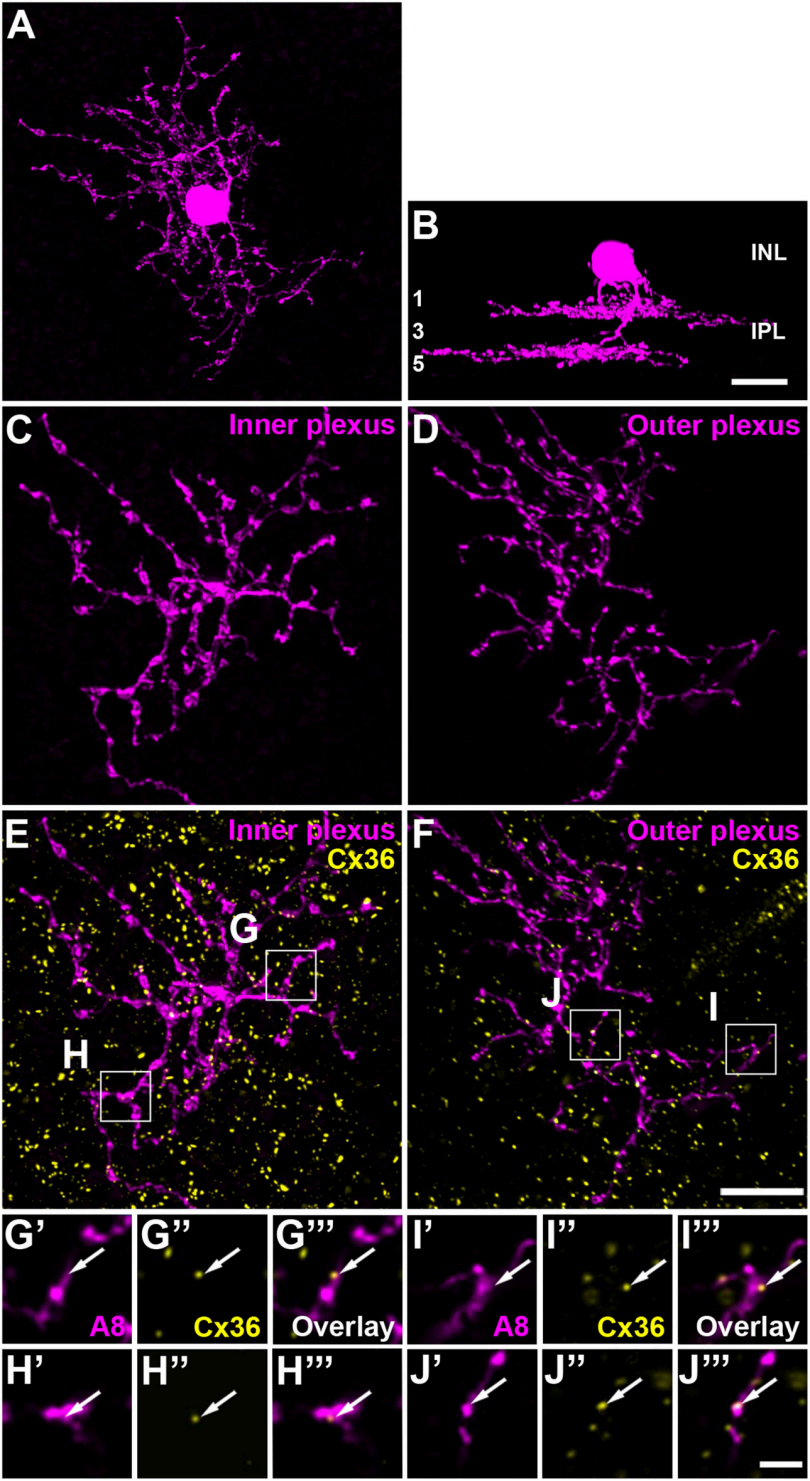
A8 cell expressed connexin36 (Cx36) at their inner and outer plexus, in the ON and OFF sublamina of the inner plexiform layer (IPL), respectively. **(A)** Maximum projection of an Alexa-568-injected A8 cell. **(B)** Same cell as in **(A)**, XZ-rotation of a confocal stack shows the typical bistratified morphology of the A8 cell. **(C,D)** Maximum projection of inner **(C)** and outer dendrites **(D)** of the injected A8 cell. **(E,F)** Maximum projection of the overlay of Cx36 with inner **(E)** and outer dendrites **(F)** of A8 cell. **(G'–J”')** Selected areas from **(E,F)** as single, magnified sections: A8 dendrites **(G',H',I',J')**, Cx36 **(G”,H”,I”,J”)**, and the respective overlays **(G”',H”',I”',J”')**. Arrows denote colocalization of Cx36 with A8 cell dendrites. Scale bar: **(A–F)**, 10 μm; **(G'–J”')**, 2 μm.

Due to the great amacrine cell diversity, it seems likely that other amacrine cells may also form electrical synapses (gap junctions) with bipolar cells, thereby potentially impacting bipolar cell signaling. Recently, we described such an amacrine cell in the mouse retina, the A8 amacrine cell (Lee et al., 2015). It shares interesting features with AII cells because it not only forms gap junctions with ON cone bipolar cells and potentially amacrine cells, but also represents a glycinergic small-field amacrine cell with bistratified morphology: OFF dendrites stratify in sublamina 1 of the IPL and ON dendrites in sublamina 4 (Figure 1). A8 cells were shown to receive glutamatergic input from OFF and ON cone bipolar cells and provide glycinergic inhibition to OFF cone bipolar cells and ON-α ganglion cells (Lee et al., 2015). However, electrical synapses of A8 cells have not been studied in detail so far. Therefore, we used a combination of intracellular dye/tracer injections, pharmacology and immunohistochemical labeling to determine the gap junction protein (connexin subunit) expressed by A8 cells and the potential coupling partners and also to discern whether A8 gap junctions are modulated by dopamine. Here, we provide evidence that the electrical coupling of A8 amacrine cells is much weaker than that of AII amacrine cells, but involves the same gap junction protein, connexin36 (Cx36). However, whereas AII coupling is plastic and modulated by dopamine (Kothmann et al., 2009) in the mouse retina, A8 coupling is not affected by activating or blocking D_1_ dopamine receptors. Furthermore, our data suggests that A8 cells are coupled to type 6 and 7 ON bipolar cells, which also represent the major coupling partners of AII amacrine cells (Tsukamoto and Omi, 2017). Interestingly, A8 cells are coupled to OFF bipolar cells as well. Taken together, our data provides evidence that electrical coupling in the inner plexiform layer can differ considerably among different cell types, even if the cells couple to the same synaptic partners.

## Materials and Methods

### Animal and Tissue Preparation

All procedures and experiments conducted in this study complied with the guidelines of the European Communities Council Directive (86/609/EEC) and the laws of the Federal Government of Germany *(Tierschutzgesetz*; BGBl. I S. 1206, 1313 and BGBl. I S. 1934) for experimental animals and were approved by the local animal care committee (*Niedersaechsisches Landesamt fuer Verbraucherschutz und Lebensmittelsicherheit*).

In this study, C57BL/6J and Ier5-EGFP mice (Siegert et al., 2009; Lee et al., 2015) were used. Mice picked for the experiments were typically 2–9 months old and of either sex.

For AII and A8 cell injections in retinal whole-mounts, mice were deeply anesthetized with CO_2_ and euthanized by cervical dislocation. Thereafter, eyes were enucleated; subsequently, cornea, lens and vitreous body were removed in extracellular solution (in mM: 110 NaCl, 2.5 KCl, 1 CaCl_2_, 1.6 MgCl_2_, 10 glucose, 22 NaHCO_3_, adjusted to pH 7.4 by gassing with 95% O_2_ and 5% CO_2_) at room temperature. Retinas were carefully extracted from the eyecups and bisected with a clean razor blade. Any residual vitreous body was thoroughly removed with a pair of blunt tweezers. Retinal pieces were then mounted on a black filter paper ganglion cell side up (Meyer et al., 2018). To visualize AII amacrine cells, the retinal piece was incubated in 0.0001% DAPI solution for 30–45 min prior to the injection.

For slice immunohistochemistry, the cornea was cut along the ora serrata, leaving the posterior eyecup which was fixed in 2% paraformaldehyde (PFA) in 0.1 M phosphate buffer (PB) for 3 × 10 min, washed in PB and immersed in 30% sucrose in PB overnight.

### Intracellular Dye and/or Neurobiotin Injections

Intracellular injections were performed as described previously (Tetenborg et al., 2017). In short, thin-walled borosilicate glass electrodes with filament were pulled with a Sutter P-97 puller (Sutter, Novato, CA, USA), resulting in electrodes with a resistance between 100 and 150 MΩ. The electrode tips were then filled with 2 μl of 5 mM Alexa Fluor 488/568 potassium hydrazide and 4% Neurobiotin (w/v), which was diluted in 0.2 M KCl. Thereafter, the electrodes were backfilled with 8 μl of 0.2 M KCl. The dye was iontophoresed with −0.5 nA square pulses (500 ms at 1 Hz) for 5 min.

In case of tracer coupling experiments, retinas were either pre-treated with 10 μM D_1_ receptor antagonist Schering23390 (Sch23390), 10 μM D_1_ receptor agonist SKF38393 (SKF) or with extracellular solution (control) for 15 min (Hampson et al., 1992). The retina was constantly superfused with either Sch23390, SKF or control solution until fixation. The Alexa dye was iontophoresed with −0.5 nA pulses for 1 min to judge the proper impalement of the amacrine cell. Subsequently, the current was reversed in order to inject Neurobiotin for 5 min. In order to minimize bias, at least a couple of cells were injected in both control and treated condition on a single day. In all cases, the dye or tracer was allowed to diffuse for 15 min ahead of fixation. Since only light-adapted retinas were used for injections under epifluorescence (1.6 W/m^2^) and in the presence of dim room light (5.5 mW/m^2^), results represent photopic conditions.

### Immunohistochemistry

The dye- or Neurobiotin-injected whole-mount retinas were fixed with 4% PFA in 0.1 M PB. Subsequently, the retinas were thoroughly washed 3 × 10 min with PB. For coupling studies, the retina was then incubated in Alexa Fluor 546/647-conjugated streptavidin (1:250) overnight at room temperature to visualize the Neurobiotin. For immunostainings, retinas were incubated with primary antibodies (in PB containing 5% NDS, 0.05% NaN_3_, 0.3% Triton-X) for 2–3 days at room temperature (Table 1). Thereafter, the samples were extensively washed and incubated with the respective secondary antibodies, conjugated to Alexa 488, Alexa 568, or Alexa 647 (1:250, Fisher Scientific), for 1 day at room temperature. The retinas were again washed thoroughly and mounted in Vectashield.

**Table 1:** List of primary antibodies used in this study.

**Antibody**	**Host, type**	**Dilution**	**Source Cat., No**.	**Citation**
CtBP2	Rabbit, polyclonal	1:1,000	Synaptic Systems, 193003	Hübler et al., 2012
Cx36	Mouse, monoclonal	1:250/1:500 (slice)	Invitrogen, 37–4600	Hilgen et al., 2011
Cx36	Rabbit, polyclonal	1:250/1:500 (slice)	Invitrogen, 36–4600	Kihara et al., 2009
Cx45	Rabbit, polyclonal	1:250	Pineda	Dedek et al., 2006
D_1_ receptor	Mouse, monoclonal	1:250	Millipore, AB5290	Farshi et al., 2016
GABA_A_-Rα3	Rabbit, polyclonal	1:1,000 (slice)	Sigma, G4291	Gao et al., 1993
GFP	Chicken, polyclonal	1:500	Millipore, AB16901	Saino-Saito et al., 2007
SCGN	Sheep, polyclonal	1:1,000/1:1,000 (slice)	BD Biosciences, 554002	Puthussery et al., 2011
Syt2	Mouse, monoclonal	1:200	Zebrafish International Resource Center	Fox and Sanes, 2007
TH	Mouse, monoclonal	1:500/1:1,000 (slice)	Immunostar, 22941	Moreno et al., 2010
VGluT1	Guinea pig, polyclonal	1:1,000	Millipore, AB5905	Hilgen et al., 2011

*Slice, concentration used for retinal cryosections*.

For immunostainings of vertical retina slices, the cryoprotected tissue was sliced into 20 μm sections. After blocking with 10% normal donkey serum (NDS) in TBS-Tx (TRIS-buffered saline with 0.3% TritonX-100, pH 7.6), the sample was incubated with primary antibodies (Table 1) diluted in blocking solution overnight (at 4°C). Secondary antibodies were conjugated to Alexa 488, Alexa 568 or Alexa 647 (1:500, Fisher Scientific) and were applied for 2 h at room temperature. Washing steps were performed exclusively with TBS-Tx.

### Image Acquisition and Analysis

Images were obtained with a confocal laser scanning microscope (Leica TCS SP8). Z-stack images of retinal whole-mounts and cryosections were acquired with HC PL APO CS2 40x/1.3 and HC PL APO CS2 63x/1.4 oil objectives, respectively. For colocalization analyses, the pixel size was kept constant (47 × 47 nm) throughout a series of experiments and the stacks were taken at a z-distance of maximally 0.2 μm between optical sections. Notably, the OFF and ON dendrites of A8 cell were scanned separately to ensure that the entire dendritic field was captured without having to compromise with resolution. To analyze A8 and AII cell coupling, the stacks were acquired at a z-distance of 0.3–0.5 μm. It should be noted that sometimes scans with higher gain were necessary in order to not miss the weakly streptavidin-stained cells in coupling experiments.

Except for the coupling data, each acquired stack was deconvolved. This was achieved by using a theoretical point spread function in Huygens Essential deconvolution software. The deconvolved data thus obtained was subsequently processed in Fiji (https://fiji.sc/, Schindelin et al., 2012) as described before (Tetenborg et al., 2017). Stacks of the injected cells were 3D-projected to visualize the stratification pattern in the IPL. A8 cells showed the typical bistratified morphology (Figure 1) (Kolb et al., 1981; Lee et al., 2015).

Colocalization between two channels was crudely estimated by the *colocalization highlighter* plugin and global thresholds (*IsoData, Otsu*, and *Moments*) in Fiji. This resulted in 8-bit and RGB images, displaying colocalized puncta of the two-tested channels, which were maximum-projected. Thereafter, the merged stack was 3D-projected and rotated 360° in x- and y-axis at 5° steps (Supplementary Movie). Simultaneously, the above mentioned colocalized puncta were tested for their association at each step. If they dissociated at any step, the colocalization was discarded. Else, the colocalization was declared to be true. As colocalization was exclusively quantified for deconvolved images, it might be slightly underestimated. For multiple channels, the colocalization analysis was conducted sequentially. Images that represent this analysis have been adjusted for brightness and contrast in Adobe Photoshop CS5 for presentation purposes.

It should be noted that the colocalization analysis was performed solely on the retinal whole-mount data. Retinal slices were only used to confirm the presence of colocalization observed in the whole-mount data. Accordingly, we did not quantify the colocalization observed in the retinal slices. We rather judged the colocalization based on the normalized pixel intensity profile of the channels of interest (Kántor et al., 2017). Also, the number of coupled cells were counted manually. Occasionally, we had to count cells at slightly higher gamma values in order to not miss weakly coupled or stained cells.

### Statistical Analysis

Unless stated otherwise, data is presented as mean ± sd (standard deviation). Data on A8 and AII coupling was not normally distributed. Therefore, the Wilcoxon rank sum test was used in Matlab R2016a to test for significant differences between control, Sch23390 and SKF at a significance level of 5%.

## Results

### Composition of Gap Junctions in A8 Amacrine Cells

The glycinergic, bistratified A8 amacrine cell (Figures 1A,B) was shown to couple to bipolar cells (mouse: Lee et al., 2015) and bipolar and amacrine cells (cat: Kolb and Nelson, 1996). However, the gap junction protein that mediates this coupling has not been identified so far. To answer this question, we dye-injected individual A8 amacrine cells in the Ier5-GFP mouse line and labeled them for Cx36 (Figures 1E–J”') and Cx45 (Figure S1), which represent the two most abundant connexins in the IPL of the mouse retina (Feigenspan et al., 2001; Dedek et al., 2006; Li et al., 2008). Injected A8 cells expressed 7.5 ± 1.8 Cx36-positive puncta on ON (Figures 1E,G–H”') and 9.9 ± 2.1 Cx36-positive puncta on OFF dendrites (Figures 1F,I–J”'; Table 2; N = 21 cells, from 15 mice). The number of colocalized puncta was rather low when compared to AII amacrine cells which were shown to express ~145 Cx36-positive puncta on their arboreal (ON) dendrites (Meyer et al., 2014). Next, we tested for Cx45 expression; however, colocalization was rare with only 2.2 ± 0.4 Cx45-positive puncta on A8 ON dendrites and 4.6 ± 1.1 Cx45-positive puncta on A8 OFF dendrites (*N* = 5 cells, from 5 mice; Figure S1). Thus, we conclude that the vast majority of A8 gap junctions are assembled from Cx36.

**Table 2:** Statistics for Cx36-containing gap junctions on the dendrites of A8 cells and the colocalization with bipolar cell terminals.

	**Cx36 ON dendrites**	**Cx36 OFF dendrites**	**VGluT1 + Cx36 ON**	**VGluT1 + Cx36 OFF**	**SCGN + CX36 ON**	**SCGN + CX36 OFF**
Colocalized	7.5 ± 1.8	9.9 ± 2.1	4.5 ± 1	4 ± 0.5	2.4 ± 0.4	1.8 ± 0.4
%			57.5 ± 12.9	46.8 ± 12	28.2 ± 3	17 ± 5.6
No. of cells	21	21	6	5	6	6
No. of mice	15	15	5	4	5	5

*Values are given as mean ± sd. % refers to the amount of Cx36 puncta on A8 ON/OFF dendrites colocalized with either VGluT1 or SCGN*.

### Coupling Partners of A8 Amacrine Cells

**Figure 2:**
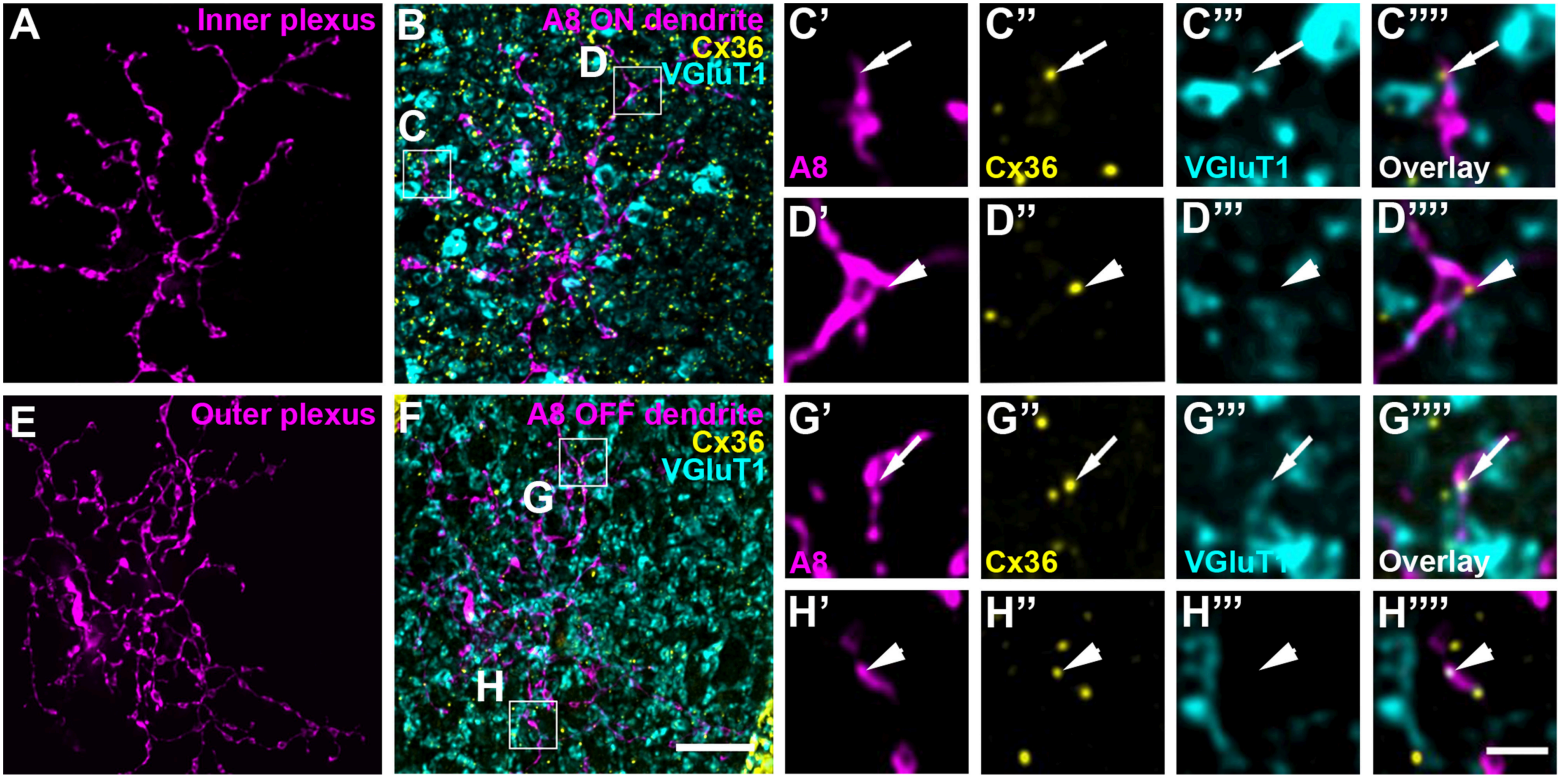
A8 cell dendrites form Cx36-containing gap junctions with ON and OFF bipolar cell terminals. **(A,E)** Maximum projection of inner **(A)** and outer A8 dendrites **(E)**. **(B,F)** Maximum projections of the overlay of Cx36 and VGluT1 with inner **(B)** and outer dendrites **(F)** of an injected A8 cell. **(C'–H”')** Selected areas from **(B,F)** as single, magnified sections: A8 dendrites **(C',D',G',H')**, Cx36 **(C”,D”,G”,H”)**, VGluT1 **(C”',D”',G”',H”')** and their respective overlay **(C”',D”',G”',H”')** within a single section from the selected ROI. Arrows denote colocalization of all three channels **(C'–C””,G'–G””)**. Arrowheads point to Cx36-positive puncta only colocalized with the injected A8 dendrite but not with VGluT1-positive bipolar cell terminals **(D'–D””,H'–H””)**. Scale bars: **(A,B,E,F)**, 10 μm; **(C'–D”')**, **(G'–H””)**, 2 μm.

**Figure 3:**
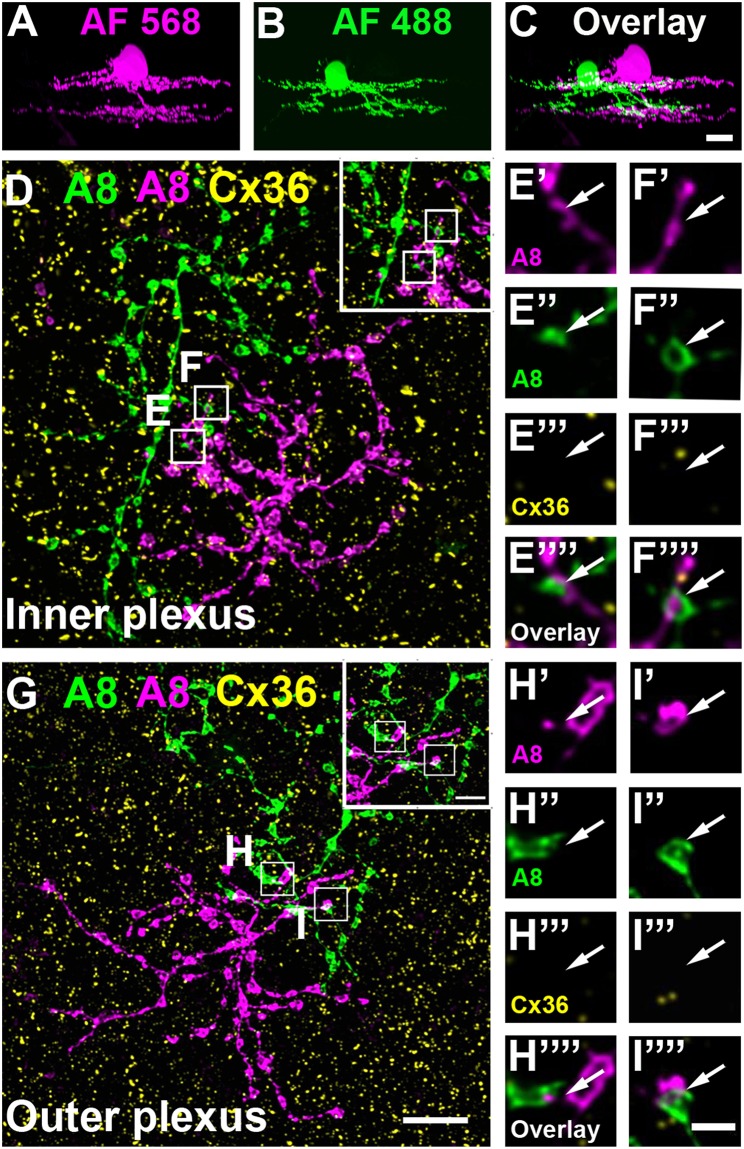
A8 amacrine cells do not contact other A8 cells via Cx36. **(A–C)** XZ rotation of two A8 cells injected with Alexa Fluor 568 **(A)** and 488 **(B)** with overlapping dendrites **(C)**. **(D–I””)** Maximum projection of inner **(D)** and outer plexus **(G)** of the A8 cell pair, stained for Cx36. Insets show enlarged maximum projections of regions with A8-A8 contacts. Boxes denote the magnified regions of interest from the inner **(E,F)** and outer plexus **(H,I)** as shown in **(E'–F”')** and **(H'–I””)**. No Cx36 labeling was detected at A8-A8 contact points (arrows). Similar results were obtained for three other A8 cell pairs. Scale bar: **(A–D,G)**, 10 μm; insets in **(D,G)**, 5 μm; **(E'–F””)**; **(H'–I””)**, 2 μm.

To test whether murine A8 cells form gap junctions only with bipolar cells (as suggested by Lee et al., 2015) or also with amacrine cells (as suggested for the cat, Kolb and Nelson, 1996), we dye-injected A8 cells in the Ier5-GFP mouse line and stained the retinal whole-mounts for Cx36 and VGluT1, which labels all bipolar cell terminals in the mouse retina (Figure 2). On both, ON (Figures 2A–C””) and OFF dendrites of A8 cells (Figures 2E–G””), we found Cx36-immunoreactive puncta between adjacent A8 processes and VGluT1-labeled axon terminals of bipolar cells. Similar results were obtained for vertical cryosections from Ier5-GFP mice labeled for GFP, VGluT1 and Cx36 (Figures 5A,D). Additionally, we found Cx36 puncta on injected A8 ON (Figures 2A,B,D'–D””) and OFF dendrites (Figures 2E,F,H'–H””) that were *not* associated with VGluT1-stained bipolar cell terminals, suggesting that these puncta represent gap junctions between A8 cells and other amacrine cells. Quantification of the colocalized puncta revealed that A8 cells bestow 57.5 ± 12.9% (N = 6 cells, from 5 mice) of their Cx36-containing gap junctions in the ON IPL and 46.8 ± 12% (N = 5 cells, from 4 mice) in the OFF IPL to bipolar cells (Table 2). This suggests that roughly half of all Cx36-positive puncta on A8 amacrine cells are involved in their coupling to bipolar cells, whereas the other half presumably serves A8-to-amacrine cell coupling. To discern whether these cells represent other A8 cells, as suggested for the cat retina (Kolb and Nelson, 1996), we dye-injected two adjacent A8 cells and labeled them for Cx36. The pairs of A8 cells showed enough dendritic overlap to allow assessing the presence or absence of Cx36 colocalization. As shown in Figure 3, we did not find Cx36 at contact points between the two cells, suggesting that A8 cells lack homologous coupling in the mouse retina.

**Figure 4:**
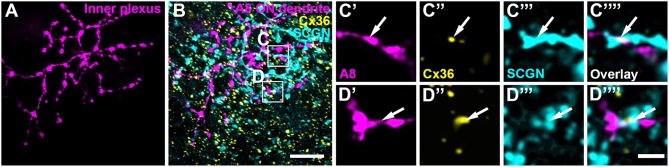
Cx36 puncta on A8 ON dendrites colocalized with secretagogin (SCGN). **(A)** Maximum projection of A8 ON dendrites. **(B)** Maximum projection of the overlay of Cx36 and SCGN with the ON dendrites of an injected A8 cell. **(C,D)** Selected ROI from B. **(C'–D””)** Magnified images of A8 dendrites **(C',D')**, Cx36 **(C”,D”)**, SCGN **(C”',D”')** and their respective overlay **(C””,D””)** within a single section from the selected ROI. Arrows denote colocalization of all three channels. Scale bar: **(A,B)**, 10 μm; **(C'–D””)**, 2 μm.

**Figure 5:**
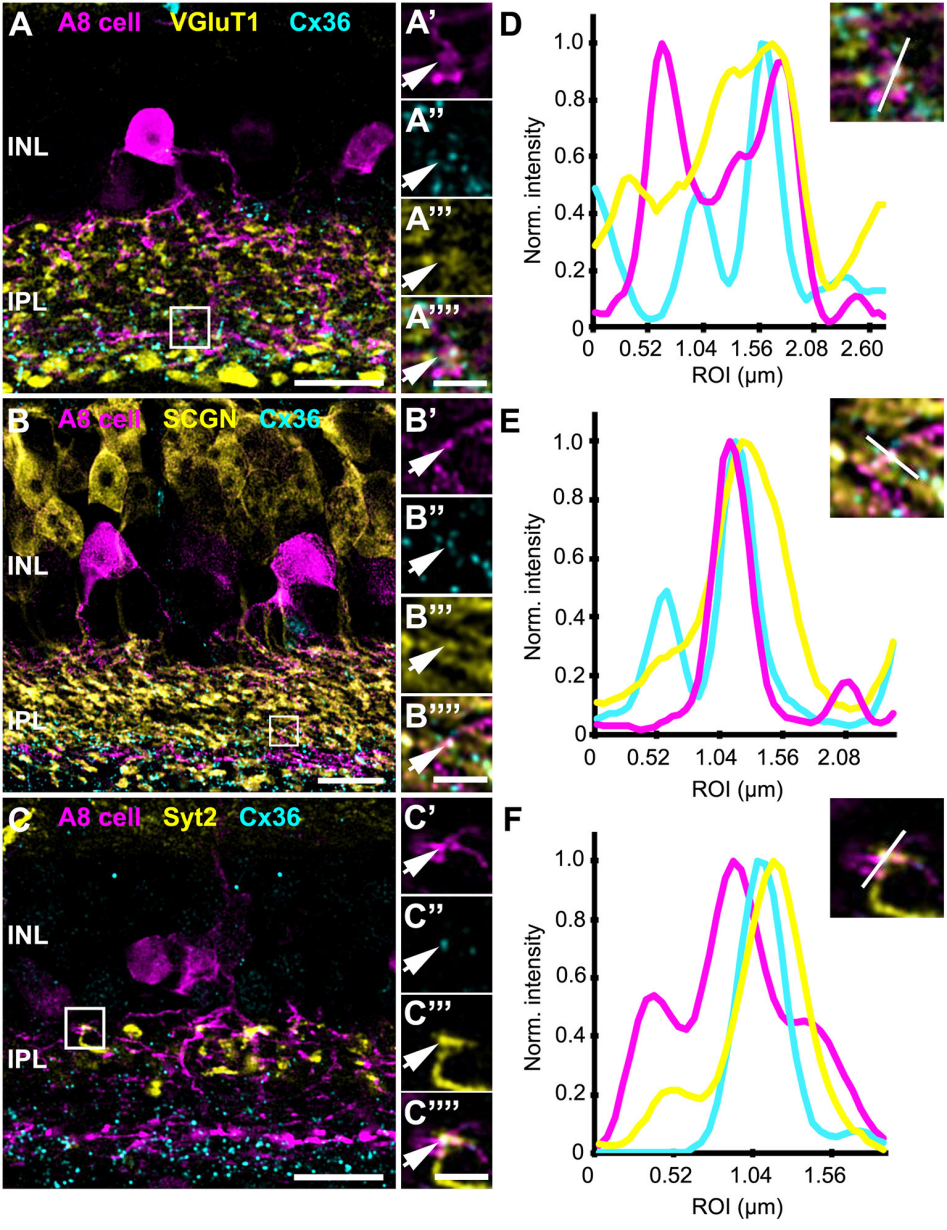
Co-localization of A8 gap junctions with bipolar cell terminals in vertical sections. **(A–C)** Single retinal slices of Ier5-EGFP (A8 cell) mouse stained with Cx36 and bipolar cell markers: VGluT1 **(A)**, secretagogin [**(B)**, SCGN], and synaptotagmin-2 [**(C)**, Syt2]. Square white boxes in **(A–C)** are the selected ROIs shown in **(A'–C””)**. Arrows denote co-localization of all the three channels which is also represented in the normalized intensity plots **(D–F)**. **(D–F)** Intensity plot for three channels, corresponding to **(A–C)**. The respective inset represents the single scan overlay of the three channels. The plot denotes normalized pixel intensity of three channels in y-axis, and the x-axis represents the relative distance of peak intensities of the three individual channels. Scale bar: **(A–C)**, 10 μm; **(A'–C””)**, 2.5 μm.

Our previous results (Lee et al., 2015) showed that A8 amacrine cells likely receive glutamatergic input from any bipolar cell that stratifies in layers 1 and 4 of the IPL: type 1 and 2 OFF bipolar cells and type 6 and 7 ON bipolar cells. Thus, our next goal was to determine the types of bipolar cells A8 cells may form gap junctions with. Out of the four potential partners, only type 2 and 6 bipolar cells express the calcium-binding protein secretagogin (SCGN, Puthussery et al., 2010). We made use of this type-specific expression and stained dye-injected A8 cells (Figure 4A) and GFP-expressing A8 cells (Figure 5B) for Cx36 and SCGN in whole-mount Figures 4B–D and slice preparations, respectively. We found Cx36-immunoreactive puncta between A8 amacrine and SCGN-positive bipolar cell dendrites in the ON IPL (Figures 4B–D””, 5B,E). These puncta comprised 28.2 ± 3% of all Cx36-positive structures on A8 dendrites in the ON IPL and thus ~49% of the gap junctions between A8 and ON cone bipolar cells (Table 2). Therefore, it seems reasonable to conclude that A8 cells form approximately the same number of gap junctions with SCGN-positive type 6 and SCGN-negative type 7 bipolar cells.

In contrast, Cx36-immunoreactive puncta between A8 dendrites and SCGN-positive OFF bipolar cell dendrites were found less frequently (Table 2). SCGN-positive OFF bipolar cells likely represent type 2 cells, which can also be labeled by antibodies directed against synaptotagmin-2 (Syt2). Although we again occasionally detected Cx36 between Syt2-positive bipolar cell dendrites and A8 cell dendrites (Figures 5C,F), the low degree of colocalization (17 ± 5.6%, *N* = 6 cells, from 5 mice) argues against substantial coupling between type 2 OFF bipolar and A8 amacrine cells. Conversely, our data suggests that A8 cells are coupled to SCGN-negative type 1 bipolar cells in OFF IPL (Figure S2), the only other bipolar cell stratifying in sublamina 1 of the IPL.

Thus, in contrast to the non-selective glutamatergic input from bipolar cells, A8 amacrine cells seem to be more specific in their coupling pattern, with almost equal coupling to type 1 OFF bipolar cells and type 6 and 7 ON bipolar cells and weaker coupling to type 2 OFF bipolar cells. These results are in line with the tracer coupling experiments in Lee et al. (2015), which showed coupling to SCGN-positive and -negative bipolar cell somata.

### Input From Dopaminergic Amacrine Cells

**Figure 6:**
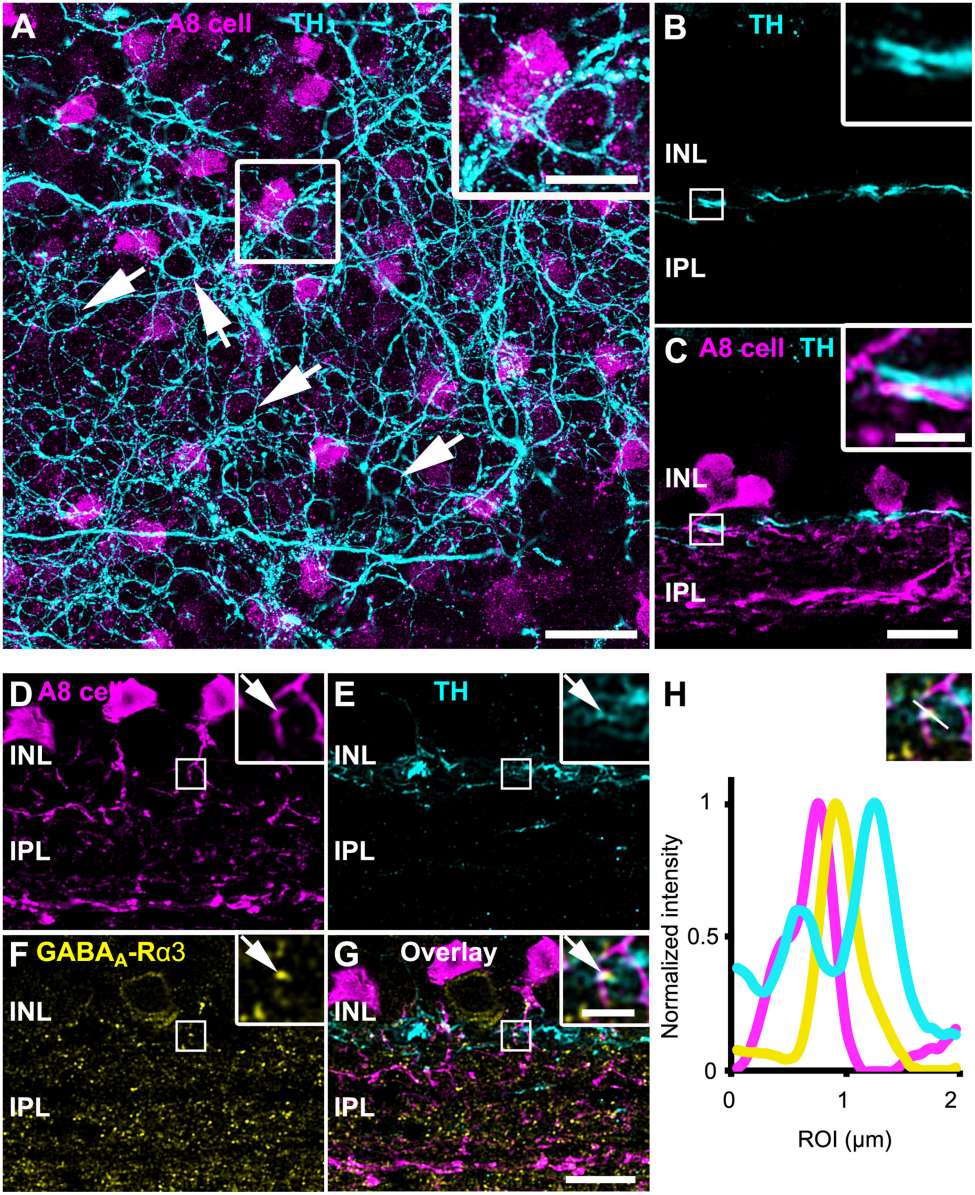
A8 cells presumably receive GABAergic input from tyrosine hydroxylase (TH)-positive cells in the OFF IPL. **(A)** Maximum projection of an Ier5-GFP whole-mount retina, labeled for TH (cyan). The inset shows the magnified view of the area marked in **(A)**. Arrows point to perisomatic TH-positive rings, surrounding putative AII cells (Debertin et al., 2015) but not A8 cells (magenta). **(B,C)** Same staining as in **(A)** in a single scan of a vertical slice. Please note the prominent co-fasciculation of TH- and GFP-positive dendrites of dopaminergic and A8 amacrine cells, respectively. **(D–G)** Maximum projection of GFP-labeled A8 cells **(D)**, TH-stained ier5-GFP retina **(E)**, GABA_A_-R α3 staining **(F)** and their overlay **(G)**. The upper inset shows the magnified view. Arrows point to the colocalization **(G)** of the three channels **(D–F)**. **(H)** Intensity profile of the colocalized area, indicating GABAergic input from dopaminergic amacrine cells to the glycinergic A8 cell. The upper inset shows the single scan of the magnified area shown in the inset of **(G)**. Scale bar: **(A)**, 20 μm; inset: 10 μm; **(B–G)**, 10 μm; inset, 2.5 μm.

As gap junctions can be modulated by dopamine (Teranishi et al., 1983) and an earlier study reported input from dopaminergic amacrine cells (DACs) to A8 cells in the cat retina (Kolb et al., 1991), we labeled GFP-expressing A8 cells in the Ier5-GFP mouse line for tyrosine hydroxylase (TH), the key enzyme in dopamine synthesis. Labeling was performed in whole-mounts (Figure 6A) and vertical cryosections (Figure 6B). In retinal whole-mounts (Figure 6A), the characteristic network of DAC dendrites became visible: many perisomatic rings were discernible, which were shown to surround the somata of AII and non-AII amacrine cells (Debertin et al., 2015). However, although the vertical section (Figures 6B,C) showed clear co-fasciculation of A8 and DAC dendrites, perisomatic rings are evidently not surrounding GFP-labeled A8 somata (Figure 6A). As DAC are also GABAergic (Contini and Raviola, 2003), we tested for GABA receptor expression at contact sites between A8 amacrine cells and DAC. Indeed, we found colocalization of all three markers in vertical sections (Figures 6D–H), suggesting that DAC may provide direct GABAergic input to A8 cells via GABA_A_-α3 receptors. However, as earlier reports identified GABA_A_-α3 receptors also on DAC (Newkirk et al., 2013), we cannot completely exclude the possibility that the GABA receptors found here are situated on the DAC side and may not represent postsynaptic receptors on A8 cells.

### Regulation of A8 Coupling by Dopamine?

**Figure 7:**
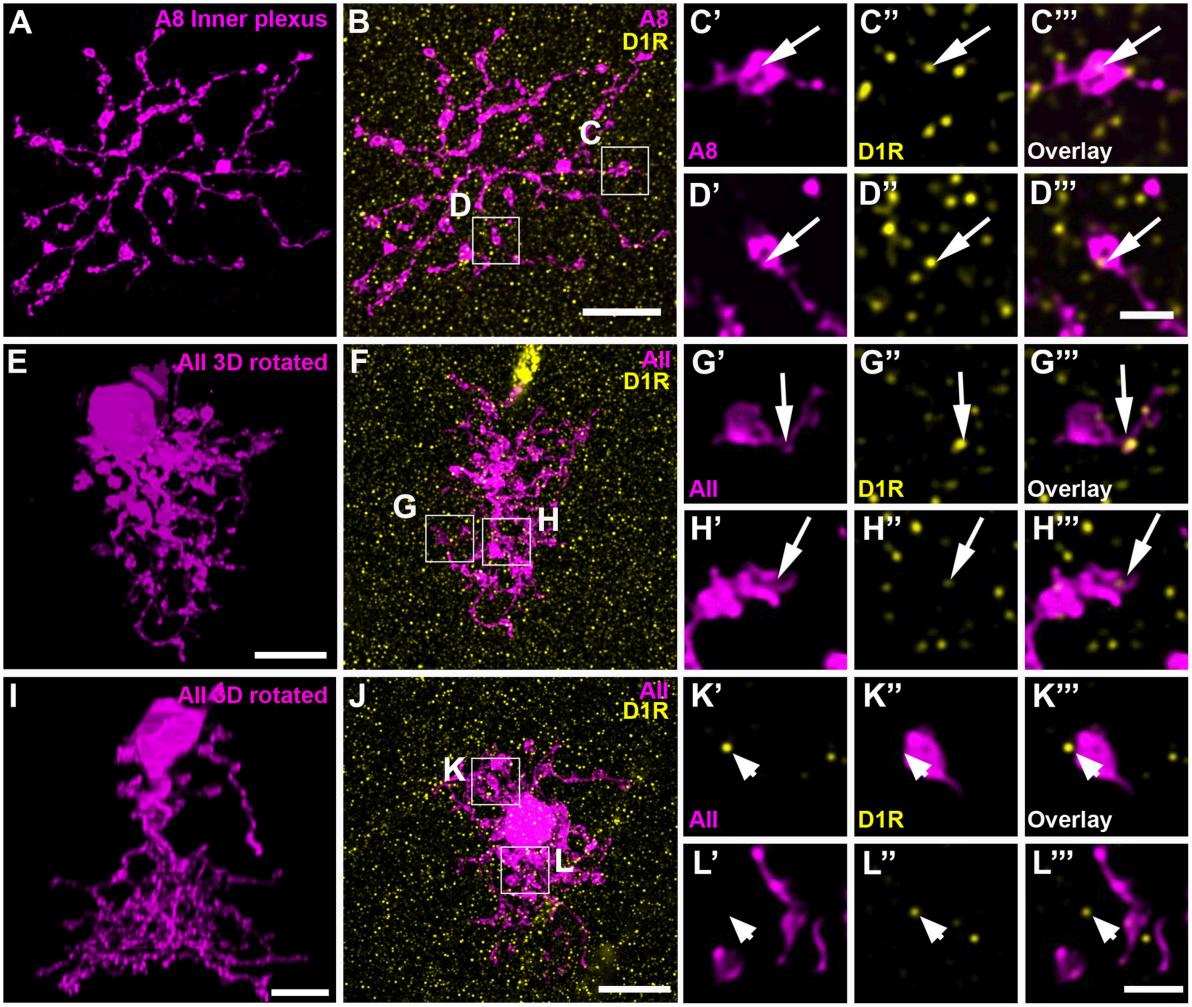
D_1_ receptors colocalized with the ON dendrites of A8 cell and most but not all AII cells. **(A)** Maximum projection of the inner dendrites of a dye-injected A8 cell. **(B)** Overlay of the A8 ON dendrites and D_1_ receptors (D1R), shown as maximum projection. **(C,D)** Selected ROI from B. **(C'–D”')** Magnified images of A8 dendrites **(C',D')**, D_1_ receptors **(C”,D”)**, and their respective overlay **(C”',D”')** within a single section from the selected ROI. Arrows denote colocalization of D_1_ receptors with A8 ON dendrites. **(E–H”')** Same as **(A–D”')** for an injected AII cell. Arrows point to D_1_ receptor immunoreactivity colocalizing with AII dendrites. **(I–L”')** Same as **(E–H”')**, however, this AII cell did not show colocalization with D_1_ receptor staining (arrowheads), consistent with a previous report on the rat retina (Veruki and Wässle, 1996). Scale bar: **(A,B,E,F,I,J)**, 10 μm; **(C'–D”')**, **(G'–H”')**, **(K'–L”')**, 2 μm.

Dopamine affects its receptor targets (D_1_ and/or D_2_ receptors) by volume transmission and diffusion (reviewed in Witkovsky, 2004). Consequently, a close contact between the DAC and A8 dendrites is not necessary for dopamine to influence A8 signaling. Therefore, we additionally tested for D_1_ and D_2_ receptor expression on dye-injected A8 cells. A8 cells did not express D_2_ receptors on their dendrites (Figure S3; Table S1). Whereas, the outer plexus was almost void of labeling, many D_1_ receptors were found on the inner plexus of A8 cells (Figures 7A–D, 11 ± 1 D_1_-positive puncta per cell, *N* = 4 cells, from 4 mice), suggesting that electrical coupling to ON cone bipolar cells may be regulated by dopamine.

**Figure 8:**
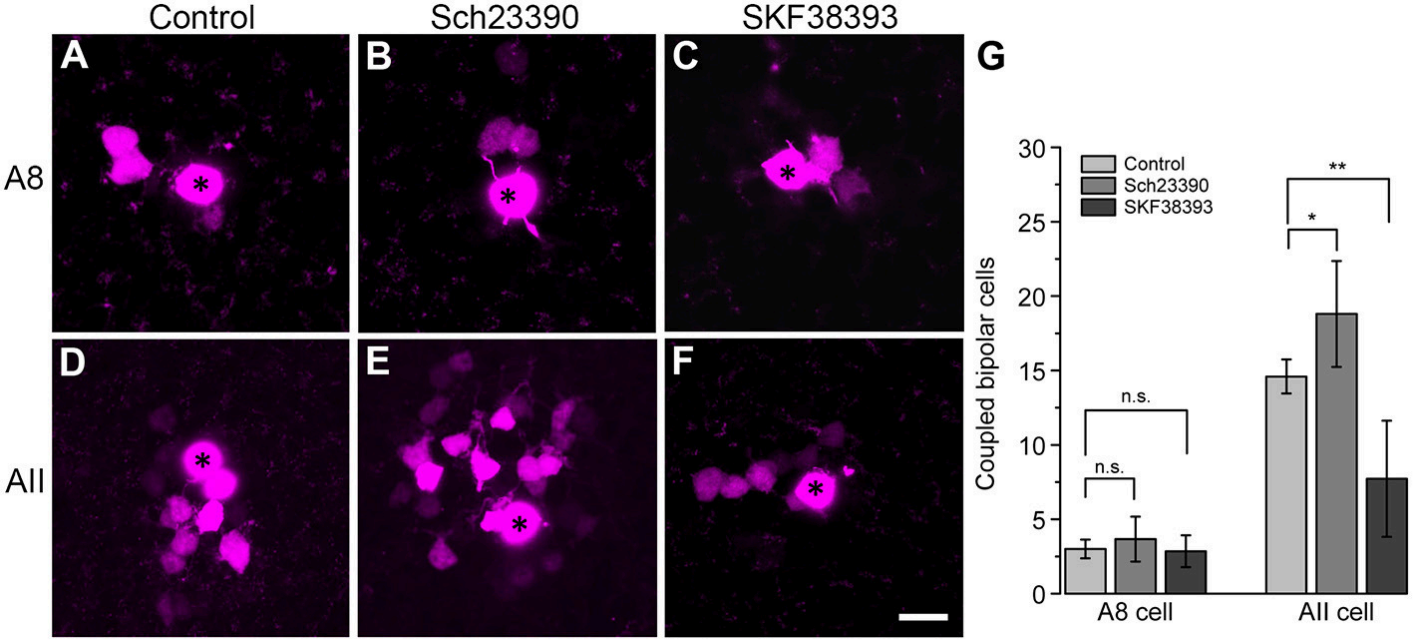
Coupling of A8 amacrine cells did not change in the presence of the D_1_ receptor antagonist Sch23390 or the D_1_ receptor agonist SKF38393. **(A,C)** Maximum projection of A8 **(A–C)** and AII amacrine cell coupling **(D–F)** to bipolar cells in control condition **(A,D)** and upon application of Sch23390 **(B,E)** or SKF38393 **(C,F)**. Asterisks denote the injected cells. Scale bar: **(A–F)**, 10 μm. **(G)** Bar graph depicting the number of bipolar cells coupled to AII and A8 cells. In AII but not A8 amacrine cells, coupling was significantly increased in the presence of the D_1_ receptor antagonist (Wilcoxon rank sum test, AII cells: *p = 0.0159, *N* = 5; A8 cells: *p* = 0.5455, *N* = 6) and significantly decreased in the presence of the D_1_ receptor agonist (Wilcoxon rank sum test, AII cells: **p = 0.0089, *N* = 7 from 5 mice; A8 cells: *p* = 0.99, *N* = 7 from 5 mice).

To test this, we performed tracer coupling experiments in ier5-GFP mice (Figure 8). Under photopic conditions, we injected A8 amacrine cells with the gap junction-impermeable dye Alexa568 and the gap junction-permeable tracer Neurobiotin (Figures 8A–C). Retinas were preincubated (15 min) and then perfused with either control extracellular solution, the D_1_ receptor antagonist Sch23390 (10 μM in extracellular solution) or the D_1_ receptor agonist SKF (10 μM in extracellular solution). Consistent with our earlier report (Lee et al., 2015), coupling of A8 cells to bipolar cells was weak under control conditions (3 ± 0.6 coupled cells). However, despite D_1_ receptor expression on A8 dendrites in the ON IPL, blocking or activating D_1_ receptors did not significantly change tracer coupling (Figure 8G, *p* = 0.5455, 3.7 ± 1.5 coupled cells upon Sch23390 application, *N* = 6 cells, from 4 mice; *p* = 0.99, 2.9 ± 1.1 coupled cells upon SKF application, *N* = 7 cells, from 5 mice). This strongly indicates that A8 coupling is not modulated by dopamine. This is surprising because A8 gap junctions are made of Cx36 which is known to be phosphorylated by a dopamine-induced signaling cascade in AII amacrine cells (Kothmann et al., 2009). However, as D_4_ receptor antibodies gave contradictory results (not shown), we cannot completely exclude that A8 gap junctions are modulated via D_4_ receptors.

To control whether our injection conditions are reliable, we also injected AII amacrine cells with and without Sch23390 in the extracellular solution (Figures 8D,E). As expected, Sch23390 significantly increased the number of bipolar cells coupled to AII cells from 14.6 ± 0.5 cells (5 cells, from 3 mice) to 18.8 ± 1.6 cells (5 cells, from 4 mice; *p* = 0.0159, Wilcoxon rank sum test). Additionally, Sch23390 incubation also increased the number of AII cells coupled to the injected AII (control: 5.6 ± 0.7 coupled cells; Sch23390: 7 ± 0.8 coupled cells). However, this effect was not significant (*p* = 0.175, Wilcoxon rank sum test, *N* = 5). Consistent with substantial expression of D_1_ receptors on most but not all AII cells (Figures 7E–L”'), SKF decreased the number of coupled bipolar cells (7.7 ± 4.4 coupled cells, N = 6 from 5 mice) compared to control (Figures 8D,F,G; *p* = 0.0089, Wilcoxon rank sum test). Please note the AII amacrine cells, like A8 cells, did not express D_2_ receptors (Figure S4).

Together, these experiments revealed that Cx36-containing gap junctions are modulated by D_1_ receptors in one amacrine cell type (AII) but not in the other (A8) although both amacrine cells form Cx36-containing gap junctions.

## Discussion

**Figure 9:**
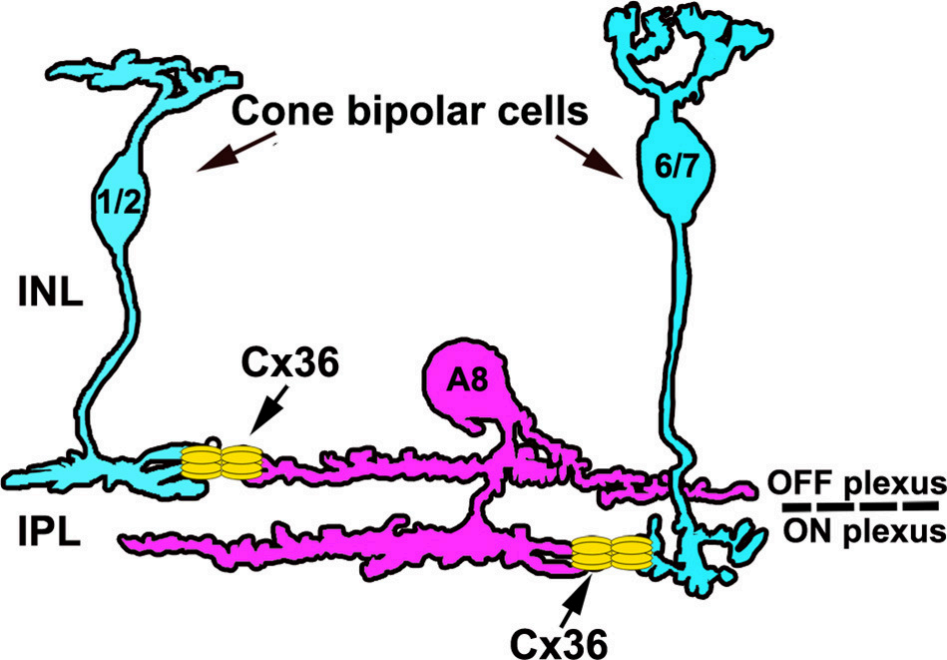
Schematic of the electrical synapses between A8 cells and cone bipolar cells. A8 cells couple to type 1 and 2 OFF bipolar and type 6 and 7 ON bipolar cells via Cx36. Please note that A8 cell dendrites also contain Cx36 puncta that are not associated with bipolar cell terminals, likely representing coupling to another amacrine cell type.

In this study, we investigated the electrical synapses of A8 cells. We show that A8 gap junctions (1) are scarce and made of Cx36, (2) provide connections to OFF and ON bipolar cells (type 1, 2, 6, and 7; Figure 9) and another cell class, (3) are only weakly tracer-permeable under photopic conditions, and (4) are not affected by the blockade or activation of D_1_ dopamine receptors (despite the abundant expression of D_1_ receptors on A8 ON dendrites). Thus, although the glycinergic bistratified A8 cell makes similar connections with bipolar cells as the rod pathway-specific AII amacrine cell, the gap junctions are very different in terms of frequency and D_1_ receptor-dependent regulation leaving their function enigmatic.

### Electrical Synapses of A8 Amacrine Cells Contain Cx36

Electrical synapses are made of connexin proteins and the different isoforms −20 in the mouse retina—differ in their electrical and biochemical properties (Söhl and Willecke, 2003), i.e., single channel conductance, sensitivity to transjunctional voltage, pH etc. Cx36 was shown to be the most abundant connexin in the mammalian retina and other brain areas; it has a low single channel conductance (9 pS, Teubner et al., 2000) and can only assemble with Cx30.2 and no other connexin into heteromeric channels (Meyer et al., 2016). Gap junctions of A8 cells are made of Cx36 and it seems likely that Cx36 is also expressed by the synaptic partners (ON and OFF bipolar cells) as we always found considerable overlap between Cx36 and the respective dendrites. Whether or not Cx36 represents the connexin in the coupled non-bipolar cells, the presumed amacrine cells (see A8 amacrine cells form gap junctions with bipolar and presumably amacrine cells), remains to be seen. As we also found weak overlap with Cx45 (Figure S1), it may also be conceivable that A8-amacrine cell coupling is heterotypic, involving Cx36 on the A8 side and Cx45 on the non-bipolar cell's side. A similar gap junction composition was suggested for AII and ON cone bipolar cells (Dedek et al., 2006).

### A8 Amacrine Cells Form Gap Junctions With Bipolar and Presumably Amacrine Cells

To shed light on the putative function of a given neuron, it is important to study its synaptic partners. Here, we show that A8 amacrine cells form gap junctions with type 1 OFF bipolar cells and type 6 and 7 ON bipolar cells, indicating that AII and A8 amacrine cells couple to common bipolar cell types (type 6 and 7, Tsukamoto and Omi, 2017). The presence of gap junctions between A8 cells and OFF bipolar cells was surprising because earlier studies (cat: Kolb and Nelson, 1996; mouse: Lee et al., 2015) and this study did not reveal any systematic tracer coupling between A8 amacrine and OFF bipolar cells. However, in one instance we discerned an OFF bipolar cell (Syt2-negative) among the coupled bipolar cells (Figure S2), indicating that the tracer is able to spread into OFF bipolar cells. In contrast, we never saw tracer coupling to other amacrine cells in the mouse retina (Lee et al., 2015; this study), although electron microscopy suggests electrical coupling among A8 cells in the cat retina (Kolb and Nelson, 1996). One potential reason for this discrepancy may be that the dendritic tree favors tracer spread into the ON arbor so that ON cone bipolar cells become easily discernible but other coupling partners do not. Similarly, in the mouse retina, homocellular AII-AII coupling is weaker compared to heterocellular AII-ON cone bipolar cell coupling (this study; Urschel et al., 2006; Meyer et al., 2014), presumably because the stout descending dendrite favors tracer spread to ON bipolar cells. Whether the A8 gap junctions that were not colocalized with VGluT1-positive bipolar cells belong to other A8 cells, other amacrine or even ganglion cells could not be discerned. However, coupling to other A8 cells seems unlikely because injecting two different dyes into adjacent A8 cells with overlapping dendrites did not reveal any Cx36-positive puncta at contact points—although we cannot completely rule out that a different connexin isoform mediates the coupling. Coupling to ganglion cells may also be unlikely because in their extensive studies on ganglion cells and Cx36, Pan et al. (2010) and Völgyi et al. (2009) did not describe a ganglion cell type that is tracer-coupled to a small bistratified amacrine cells. Thus, we conclude that A8 cells form heterocellular gap junctions with OFF and ON cone bipolar cells, and potentially gap junctions with other amacrine cells.

### Tracer Coupling of A8 Amacrine Cells Was Not Modulated by D_1_ Receptors

In recent years, evidence accumulated that gap junctions are not static but highly dynamic multi-protein complexes (reviewed in O'Brien, 2014). The neuromodulator dopamine, for example, was shown to directly influence gap junction permeability in different retinal neurons (amacrines: Hampson et al., 1992; Mills and Massey, 1995; horizontal cells: He et al., 2000). Interestingly, the Cx36-containing gap junctions of AII cells in the rabbit retina were shown to be differentially regulated: The heterocellular AII-ON cone bipolar cell gap junctions are regulated by cGMP-dependent pathways (Mills and Massey, 1995); the homocellular AII-AII gap junctions, in contrast, are closed by dopamine (Mills and Massey, 1995), via an intracellular signaling cascade leading to PP2A-mediated dephosphorylation of Cx36 (Kothmann et al., 2009). Our data show that blocking the D_1_ dopamine receptors of AII cells with Sch23390 increases the coupling to ON cone bipolar cells, consistent with a previous study on mouse retina (Urschel et al., 2006). However, this may partially represent a secondary effect because the gap junction-permeable tracer spread slightly stronger within the AII network and presumably from there to more ON cone bipolar cells (Figures 8D,E,G). Moreover, activation of D_1_ receptors by SKF decreased ON cone bipolar /AII cell coupling. In contrast, Sch23390 and SKF did not affect A8 coupling. The reason may be the rather low number of gap junction plaques mediating A8-ON cone bipolar cell and A8-amacrine cell coupling. Secondary dye spread is probably minimal, preventing an increase in coupling when D_1_ receptors are blocked. Thus, the numerous D_1_ receptors on A8 cell ON dendrites, which do not colocalize with Cx36 (Figure S5), likely affect chemical instead of electrical synapses on A8 cells. Indeed, previous work suggests that D_1_ receptors may cause changes in amacrine cell-mediated inhibition in the mouse retina (Flood et al., 2018). Please note that our study may not exclude the possibility that A8 coupling is modulated via other dopamine receptors (e.g., D_4_) or that it is modulated by NO/cGMP, as shown for ON cone bipolar/AII coupling (Mills and Massey, 1995).

### Functional Implications and Comparison With AII Amacrine Cells

A8 amacrine and AII amacrine cells share many features: (a) They are both small-field amacrine cells and consequently are densely distributed across the retina (Helmstaedter et al., 2013). (b) Both cells are bistratified and make connections with ON and OFF bipolar cells. (c) AII and A8 cells are glycinergic, providing cross-over inhibition (Werblin, 2010) to OFF bipolar and ganglion cells. However, whereas AII cells inhibit OFF-α ganglion cells (mouse: van Wyk et al., 2009), A8 cells provide glycinergic input to ON-α ganglion cells (Lee et al., 2015). (d) Both cells form electrical synapses with bipolar cells but gap junctions of A8 cells differ from that of AII cells in three major respects: (1) A8 gap junctions are less numerous (<20 per cell) compared to AII gap junctions which were shown to form ~150 per cell (Meyer et al., 2014), although a recent electron microscopy study demonstrated less gap junctions per AII cell (~50, Tsukamoto and Omi, 2017). As both cells are also coupled to non-bipolar cells, it is necessary to compare the number of gap junctions to bipolar cells between the two cell types. Of the ~17 gap junctions an A8 cell is forming, it employs ~8 to couple with bipolar cells. In comparison, AII amacrine cells were shown to use ~25–30 gap junctions for coupling with ON cone bipolar cells (Meyer et al., 2014; Tsukamoto and Omi, 2017). Both cell types couple to type 6 and 7 ON bipolar cells, but data from electron microscopy suggests that coupling to AII cells (Tsukamoto and Omi, 2017) is much stronger than to A8 cells for a given type 6 or 7 cell. 2) A8 cells also form gap junctions with OFF bipolar cells whereas AII cells only contact ON cone bipolar cells via electrical synapses (Veruki and Hartveit, 2002; Tsukamoto and Omi, 2017). This suggests that A8 cells make gap junctions with the same cells they receive glutamatergic input from, indicating that gap junctions may serve to facilitate cone bipolar cell signaling. Excitation from bipolar cells presumably reaches the A8 dendrite first via the electrical synapse, slightly depolarizing the A8 dendrite. The subsequent glutamatergic input could then be enhanced (Kolb and Nelson, 1996; Lee et al., 2015). This speculation would be bolstered by the presence of mixed synapses at A8 cells. However, we did not find any colocalization for ribbon and electrical synapses on A8 cell dendrites, despite the considerable overlap of Cx36 with CtBP2-labeled ribbons in the ON sublamina of the IPL (Table S2). The majority of these mixed synapses potentially belongs to AII cells as virtually every class of ON cone bipolar cells couples to AII cells (Tsukamoto and Omi, 2017), including SCGN-positive and -negative bipolar cells (Figure S6). (3) Electrical coupling is much weaker with only 3–4 coupled bipolar cells in A8 cells under photopic conditions, in contrast to AII cells which are tracer-coupled to ~15 bipolar cells when injected under the same conditions. (4) Finally, A8 coupling is not modulated by dopamine but potentially, other intracellular signaling cascades may affect A8 coupling. However, given the low amount of Cx36 immunoreactivity on A8 dendrites (Table 2), it seems unlikely that coupling will be strong under any light condition or activity-dependent modulation.

In summary, although A8 and AII cells share many properties, they presumably fulfill very different functions. Initially, we hypothesized that A8 cells may support AII cells in mediating rod-derived signals into the cone pathways (Güldenagel et al., 2001; Deans et al., 2002) because they may receive signals from rod bipolar cells indirectly via AII cells and ON cone bipolar cells. However, given the low degree of electrical coupling to ON cone bipolar cells, this scenario seems unlikely now. Interestingly, we found that A8 cells may provide glycinergic inhibition to the OFF dendrites of AII cells (data not shown), opening up the possibility that A8 cells directly influence the AII pathways. Collectively, our study may form the basis for further analyses elucidating the function of A8 cells in retinal processing.

## Ethics Statement

All procedures and experiments conducted in this study complied with the guidelines of the European Communities Council Directive (86/609/EEC) and the laws of the Federal Government of Germany (Tierschutzgesetz; BGBl. I S. 1206, 1313 and BGBl. I S. 1934) for experimental animals and were approved by the local animal care committee (Niedersaechsisches Landesamt fuer Verbraucherschutz und Lebensmittelsicherheit).

## Author Contributions

KD, ST, and SY designed experiments. ST and SY performed experiments. All authors contributed to the interpretation of data. KD wrote the manuscript with help from SY. ST and SY prepared all figures which were finalized by KD. All authors edited and commented on the manuscript and KD finalized it.

### Conflict of Interest Statement

The authors declare that the research was conducted in the absence of any commercial or financial relationships that could be construed as a potential conflict of interest.

## References

[B1] Bloomfield S. A. Völgyi B. (2004). Function and plasticity of homologous coupling between AII amacrine cells. Vision Res. 44, 3297–3306. 10.1016/j.visres.2004.07.012 15535997

[B2] Chen X. Hsueh H. A. Werblin F. S. (2011). Amacrine-to-amacrine cell inhibition: spatiotemporal properties of GABA and glycine pathways. Vis. Neurosci. 28, 193–204. 10.1017/S0952523811000137 21676336

[B3] Contini M. Raviola E. (2003). GABAergic synapses made by a retinal dopaminergic neuron. Proc. Natl. Acad. Sci. U.S.A. 100, 1358–1363. 10.1073/pnas.0337681100 12547914 PMC298777

[B4] Deans M. R. Volgyi B. Goodenough D. A. Bloomfield S. A. Paul D. L. (2002). Connexin36 is essential for transmission of rod-mediated visual signals in the mammalian retina. Neuron 36, 703–712. 10.1016/S0896-6273(02)01046-2 12441058 PMC2834592

[B5] Debertin G. Kántor O. Kovács-Öller T. Balogh L. Szabó-Meleg E. Orbán J.. (2015). Tyrosine hydroxylase positive perisomatic rings are formed around various amacrine cell types in the mammalian retina. J. Neurochem. 134, 416–428. 10.1111/jnc.13144 25940543

[B6] Dedek K. Schultz K. Pieper M. Dirks P. Maxeiner S. Willecke K.. (2006). Localization of heterotypic gap junctions composed of connexin45 and connexin36 in the rod pathway of the mouse retina. Eur. J. Neurosci. 24, 1675–1686. 10.1111/j.1460-9568.2006.05052.x 17004931

[B7] Farshi P. Fyk-Kolodziej B. Krolewski D. M. Walker P. D. Ichinose T. (2016). Dopamine D1 receptor expression is bipolar cell type-specific in the mouse retina. J. Comp. Neurol. 524, 2059–2079. 10.1002/cne.23932 26587737 PMC4860096

[B8] Feigenspan A. Teubner B. Willecke K. Weiler R. (2001). Expression of neuronal connexin36 in AII amacrine cells of the mammalian retina. J. Neurosci. 21, 230–239. 10.1523/JNEUROSCI.21-01-00230.2001 11150340 PMC6762459

[B9] Flood M. D. Moore-Dotson J. M. Eggers E. D. (2018). Dopamine D1 receptor activation contributes to light-adapted changes in retinal inhibition to rod bipolar cells. J. Neurophysiol. 120, 867–879. 10.1152/jn.00855.2017 29847232 PMC6139461

[B10] Fox M. A. Sanes J. R. (2007). Synaptotagmin I and II are present in distinct subsets of central synapses. J. Comp. Neurol. 503, 280–296. 10.1002/cne.21381 17492637

[B11] Gao B. Fritschy J. M. Benke D. Mohler H. (1993). Neuron-specific expression of GABAA-receptor subtypes: differential association of the alpha 1- and alpha 3-subunits with serotonergic and GABAergic neurons. Neuroscience 54, 881–892. 10.1016/0306-4522(93)90582-Z 8393540

[B12] Güldenagel M. Ammermüller J. Feigenspan A. Teubner B. Degen J. Söhl G.. (2001). Visual transmission deficits in mice with targeted disruption of the gap junction gene connexin36. J. Neurosci. 21, 6036–6044. 10.1523/JNEUROSCI.21-16-06036.2001 11487627 PMC6763178

[B13] Hampson E. C. Vaney D. I. Weiler R. (1992). Dopaminergic modulation of gap junction permeability between amacrine cells in mammalian retina. J. Neurosci. 12, 4911–4922. 10.1523/JNEUROSCI.12-12-04911.1992 1281499 PMC6575774

[B14] He S. Weiler R. Vaney D. I. (2000). Endogenous dopaminergic regulation of horizontal cell coupling in the mammalian retina. J. Comp. Neurol. 418, 33–40. 10.1002/(SICI)1096-9861(20000228)418:1<33::AID-CNE3>3.0.CO;2-J 10701754

[B15] Helmstaedter M. Briggman K. L. Turaga S. C. Jain V. Seung H. S. Denk W. (2013). Connectomic reconstruction of the inner plexiform layer in the mouse retina. Nature 500, 168–174. 10.1038/nature12346 23925239

[B16] Hilgen G. von Maltzahn J. Willecke K. Weiler R. Dedek K. (2011). Subcellular distribution of connexin45 in OFF bipolar cells of the mouse retina. J. Comp. Neurol. 519, 433–450. 10.1002/cne.22526 21192077

[B17] Hübler D. Rankovic M. Richter K. Lazarevic V. Altrock W. D. Fischer K.-D.. (2012). Differential spatial expression and subcellular localization of CtBP family members in rodent brain. PLoS ONE 7:e39710. 10.1371/journal.pone.0039710 22745816 PMC3382178

[B18] Kántor O. Varga A. Nitschke R. Naumann A. Énzsöly A. Lukáts Á.. (2017). Bipolar cell gap junctions serve major signaling pathways in the human retina. Brain Struct. Funct. 222, 2603–2624. 10.1007/s00429-016-1360-4 28070649

[B19] Kihara A. H. Paschon V. Cardoso C. M. Higa G. S. V. Castro L. M. Hamassaki D. E.. (2009). Connexin36, an essential element in the rod pathway, is highly expressed in the essentially rodless retina of *Gallus gallus*. J. Comp. Neurol. 512, 651–663. 10.1002/cne.21920 19051319

[B20] Kolb H. Cuenca N. Dekorver L. (1991). Postembedding immunocytochemistry for GABA and glycine reveals the synaptic relationships of the dopaminergic amacrine cell of the cat retina. J. Comp. Neurol. 310, 267–284. 10.1002/cne.903100210 1720142

[B21] Kolb H. Nelson R. (1996). Hyperpolarizing, small-field, amacrine cells in cone pathways of cat retina. J. Comp. Neurol. 371, 415–436. 10.1002/(SICI)1096-9861(19960729)371:3<415::AID-CNE5>3.0.CO;2-5 8842896

[B22] Kolb H. Nelson R. Mariani A. (1981). Amacrine cells, bipolar cells and ganglion cells of the cat retina: a Golgi study. Vision Res. 21, 1081–1114. 10.1016/0042-6989(81)90013-4 7314489

[B23] Kothmann W. W. Massey S. C. O'Brien J. (2009). Dopamine-stimulated dephosphorylation of connexin 36 mediates AII amacrine cell uncoupling. J. Neurosci. 29, 14903–14911. 10.1523/JNEUROSCI.3436-09.2009 19940186 PMC2839935

[B24] Lee S. C. S. Meyer A. Schubert T. Hüser L. Dedek K. Haverkamp S. (2015). Morphology and connectivity of the small bistratified A8 amacrine cell in the mouse retina. J. Comp. Neurol. 523, 1529–1547. 10.1002/cne.23752 25630271 PMC4439304

[B25] Li X. Kamasawa N. Ciolofan C. Olson C. O. Lu S. Davidson K. G. V.. (2008). Connexin45-containing neuronal gap junctions in rodent retina also contain Connexin36 in both apposing hemiplaques, forming bihomotypic gap junctions, with scaffolding contributed by zonula occludens-1. J. Neurosci. 28, 9769–9789. 10.1523/JNEUROSCI.2137-08.2008 18815262 PMC2638127

[B26] Marc R. E. Anderson J. R. Jones B. W. Sigulinsky C. L. Lauritzen J. S. (2014). The AII amacrine cell connectome: a dense network hub. Front. Neural Circuits 8:104. 10.3389/fncir.2014.00104 25237297 PMC4154443

[B27] Maxeiner S. Dedek K. Janssen-Bienhold U. Ammermüller J. Brune H. Kirsch T.. (2005). Deletion of connexin45 in mouse retinal neurons disrupts the rod/cone signaling pathway between AII amacrine and ON cone bipolar cells and leads to impaired visual transmission. J. Neurosci. 25, 566–576. 10.1523/JNEUROSCI.3232-04.2005 15659592 PMC6725315

[B28] Menger N. Pow D. V. Wässle H. (1998). Glycinergic amacrine cells of the rat retina. J. Comp. Neurol. 401, 34–46. 10.1002/(SICI)1096-9861(19981109)401:1<34::AID-CNE3>3.0.CO;2-P 9802699

[B29] Meyer A. Hilgen G. Dorgau B. Sammler E. M. Weiler R. Monyer H.. (2014). AII amacrine cells discriminate between heterocellular and homocellular locations when assembling connexin36-containing gap junctions. J. Cell. Sci. 127, 1190–1202. 10.1242/jcs.133066 24463820 PMC3953814

[B30] Meyer A. Tetenborg S. Greb H. Segelken J. Dorgau B. Weiler R.. (2016). Connexin30.2: *in vitro* interaction with Connexin36 in HeLa cells and expression in AII amacrine cells and intrinsically photosensitive ganglion cells in the mouse retina. Front. Mol. Neurosci. 9:36. 10.3389/fnmol.2016.00036 27303262 PMC4882342

[B31] Meyer A. Yadav S. C. Dedek K. (2018). Phenotyping of gap-junctional coupling in the mouse retina. Methods Mol. Biol. 1753, 249–259. 10.1007/978-1-4939-7720-8_17 29564794

[B32] Mills S. L. Massey S. C. (1995). Differential properties of two gap junctional pathways made by AII amacrine cells. Nature 377, 734–737. 10.1038/377734a0 7477263

[B33] Moreno N. Morona R. López J. M. González A. (2010). Subdivisions of the turtle Pseudemys scripta subpallium based on the expression of regulatory genes and neuronal markers. J. Comp. Neurol. 518, 4877–4902. 10.1002/cne.22493 21031557

[B34] Newkirk G. S. Hoon M. Wong R. O. Detwiler P. B. (2013). Inhibitory inputs tune the light response properties of dopaminergic amacrine cells in mouse retina. J. Neurophysiol. 110, 536–552. 10.1152/jn.00118.2013 23636722 PMC3727066

[B35] O'Brien J. (2014). The ever-changing electrical synapse. Curr. Opin. Neurobiol. 29, 64–72. 10.1016/j.conb.2014.05.011 24955544 PMC4252917

[B36] Pan F. Paul D. L. Bloomfield S. A. Völgyi B. (2010). Connexin36 is required for gap junctional coupling of most ganglion cell subtypes in the mouse retina. J. Comp. Neurol. 518, 911–927. 10.1002/cne.22254 20058323 PMC2860380

[B37] Puthussery T. Gayet-Primo J. Taylor W. R. (2010). Localization of the calcium-binding protein secretagogin in cone bipolar cells of the mammalian retina. J. Comp. Neurol. 518, 513–525. 10.1002/cne.22234 20020539 PMC3855033

[B38] Puthussery T. Gayet-Primo J. Taylor W. R. Haverkamp S. (2011). Immunohistochemical identification and synaptic inputs to the diffuse bipolar cell type DB1 in macaque retina. J. Comp. Neurol. 519, 3640–3656. 10.1002/cne.22756 22006647 PMC3500389

[B39] Saino-Saito S. Cave J. W. Akiba Y. Sasaki H. Goto K. Kobayashi K.. (2007). ER81 and CaMKIV identify anatomically and phenotypically defined subsets of mouse olfactory bulb interneurons. J. Comp. Neurol. 502, 485–496. 10.1002/cne.21293 17394138

[B40] Schindelin J. Arganda-Carreras I. Frise E. Kaynig V. Longair M. Pietzsch T.. (2012). Fiji: an open-source platform for biological-image analysis. Nat. Methods 9, 676–682. 10.1038/nmeth.2019 22743772 PMC3855844

[B41] Siegert S. Scherf B. G. Del Punta K. Didkovsky N. Heintz N. Roska B. (2009). Genetic address book for retinal cell types. Nat. Neurosci. 12, 1197–1204. 10.1038/nn.2370 19648912

[B42] Söhl G. Willecke K. (2003). An update on connexin genes and their nomenclature in mouse and man. Cell Commun. Adhes. 10, 173–180. 10.1080/cac.10.4-6.173.180 14681012

[B43] Teranishi T. Negishi K. Kato S. (1983). Dopamine modulates S-potential amplitude and dye-coupling between external horizontal cells in carp retina. Nature 301, 243–246. 10.1038/301243a0 6401844

[B44] Tetenborg S. Yadav S. C. Hormuzdi S. G. Monyer H. Janssen-Bienhold U. Dedek K. (2017). Differential distribution of retinal Ca2+/Calmodulin-Dependent Kinase II (CaMKII) isoforms indicates CaMKII-β and -δ as specific elements of electrical synapses made of Connexin36 (Cx36). Front. Mol. Neurosci. 10:425. 10.3389/fnmol.2017.00425 29311815 PMC5742114

[B45] Teubner B. Degen J. Söhl G. Güldenagel M. Bukauskas F. F. Trexler E. B.. (2000). Functional expression of the murine connexin 36 gene coding for a neuron-specific gap junctional protein. J. Membr. Biol. 176, 249–262. 10.1007/s00232001094 10931976 PMC3659790

[B46] Tsukamoto Y. Omi N. (2013). Functional allocation of synaptic contacts in microcircuits from rods via rod bipolar to AII amacrine cells in the mouse retina. J. Comp. Neurol. 521, 3541–3555. 10.1002/cne.23370 23749582 PMC4265793

[B47] Tsukamoto Y. Omi N. (2017). Classification of mouse retinal bipolar cells: type-specific connectivity with special reference to rod-driven AII amacrine pathways. Front. Neuroanat. 11:92. 10.3389/fnana.2017.00092 29114208 PMC5660706

[B48] Urschel S. Höher T. Schubert T. Alev C. Söhl G. Wörsdörfer P.. (2006). Protein kinase A-mediated phosphorylation of connexin36 in mouse retina results in decreased gap junctional communication between AII amacrine cells. J. Biol. Chem. 281, 33163–33171. 10.1074/jbc.M606396200 16956882

[B49] van Wyk M. Wässle H. Taylor W. R. (2009). Receptive field properties of ON- and OFF-ganglion cells in the mouse retina. Vis. Neurosci. 26, 297–308. 10.1017/S0952523809990137 19602302 PMC2874828

[B50] Veruki M. L. Hartveit E. (2002). Electrical synapses mediate signal transmission in the rod pathway of the mammalian retina. J. Neurosci. 22, 10558–10566. 10.1523/JNEUROSCI.22-24-10558.2002 12486148 PMC6758447

[B51] Veruki M. L. Wässle H. (1996). Immunohistochemical localization of dopamine D1 receptors in rat retina. Eur. J. Neurosci. 8, 2286–2297. 10.1111/j.1460-9568.1996.tb01192.x 8950093

[B52] Völgyi B. Chheda S. Bloomfield S. A. (2009). Tracer coupling patterns of the ganglion cell subtypes in the mouse retina. J. Comp. Neurol. 512, 664–687. 10.1002/cne.21912 19051243 PMC3373319

[B53] Werblin F. S. (2010). Six different roles for crossover inhibition in the retina: correcting the nonlinearities of synaptic transmission. Vis. Neurosci. 27, 1–8. 10.1017/S0952523810000076 20392301 PMC2990954

[B54] Werblin F. S. (2011). The retinal hypercircuit: a repeating synaptic interactive motif underlying visual function. J. Physiol. 589, 3691–3702. 10.1113/jphysiol.2011.210617 21669978 PMC3171878

[B55] Witkovsky P. (2004). Dopamine and retinal function. Doc. Ophthalmol. 108, 17–40. 10.1023/B:DOOP.0000019487.88486.0a 15104164

