# Corrigendum: Gap Junctions in A8 Amacrine Cells Are Made of Connexin36 but Are Differently Regulated Than Gap Junctions in AII Amacrine Cells

**DOI:** 10.3389/fnmol.2019.00149

**Published:** 2019-06-12

**Authors:** Shubhash C. Yadav, Stephan Tetenborg, Karin Dedek

**Affiliations:** ^1^Animal Navigation/Neurosensorics, Institute for Biology and Environmental Sciences, University of Oldenburg, Oldenburg, Germany; ^2^Research Center Neurosensory Science, University of Oldenburg, Oldenburg, Germany

**Keywords:** amacrine cell, bipolar cell, gap junction, electrical synapse, connexin36, retina, dopamine

In the original article, there was a mistake in Figure 5 as published. The line scans in Figures 5E,F depicting the channel intensity of the respective ROIs in Figures 5B,C, were swapped by mistake. The corrected Figure 5 appears below.

**Figure 5:**
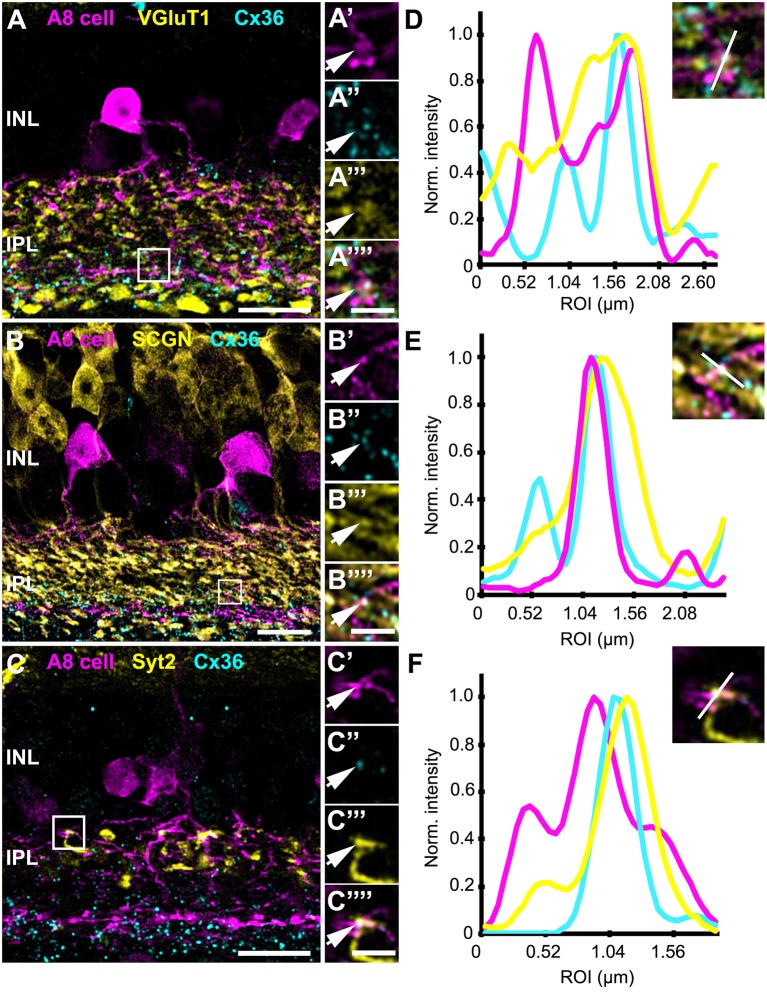
Co-localization of A8 gap junctions with bipolar cell terminals in vertical sections. **(A–C)** Single retinal slices of Ier5-EGFP (A8 cell) mouse stained with Cx36 and bipolar cell markers: VGluT1 **(A)**, secretagogin [**(B)**, SCGN], and synaptotagmin-2 [**(C)**, Syt2]. Square white boxes in **(A–C)** are the selected ROIs shown in **(A'–C””)**. Arrows denote co-localization of all the three channels which is also represented in the normalized intensity plots **(D–F)**. **(D–F)** Intensity plot for three channels, corresponding to **(A–C)**. The respective inset represents the single scan overlay of the three channels. The plot denotes normalized pixel intensity of three channels in y-axis, and the x-axis represents the relative distance of peak intensities of the three individual channels. Scale bar: **(A–C)**, 10 μm; **(A'–C””)**, 2.5 μm.

The authors apologize for this error and state that this does not change the scientific conclusions of the article in any way. The original article has been updated.

