# Supplementary material for: Gap Junctions in A8 Amacrine Cells Are Made of Connexin36 but Are Differently Regulated Than Gap Junctions in AII Amacrine Cells
Source: Front Mol Neurosci. 2019 Apr 23;12:99. doi: 10.3389/fnmol.2019.00099 (PMC6489437; doi:10.3389/fnmol.2019.00099)
Supplement: Supplementary file 1 [file Data_Sheet_1.pdf]

Supplementary Material

Gap Junctions in A8 Amacrine Cells Are Made of Connexin36 but Are Differently Regulated than Gap Junctions in AII Amacrine Cells

Shubhash C. Yadav[1], Stephan Tetenborg[1], Karin Dedek[1,2*]

[1]Animal Navigation/Neurosensorics, Institute for Biology and Environmental Sciences, University of Oldenburg, Oldenburg, Germany
[2]Research Center Neurosensory Science, University of Oldenburg, Oldenburg, Germany

***Correspondence:**
Karin Dedek
karin.dedek@uni-oldenburg.de

1 Supplementary Data

2 Supplementary Figures and Tables

2.1 Supplementary Figures

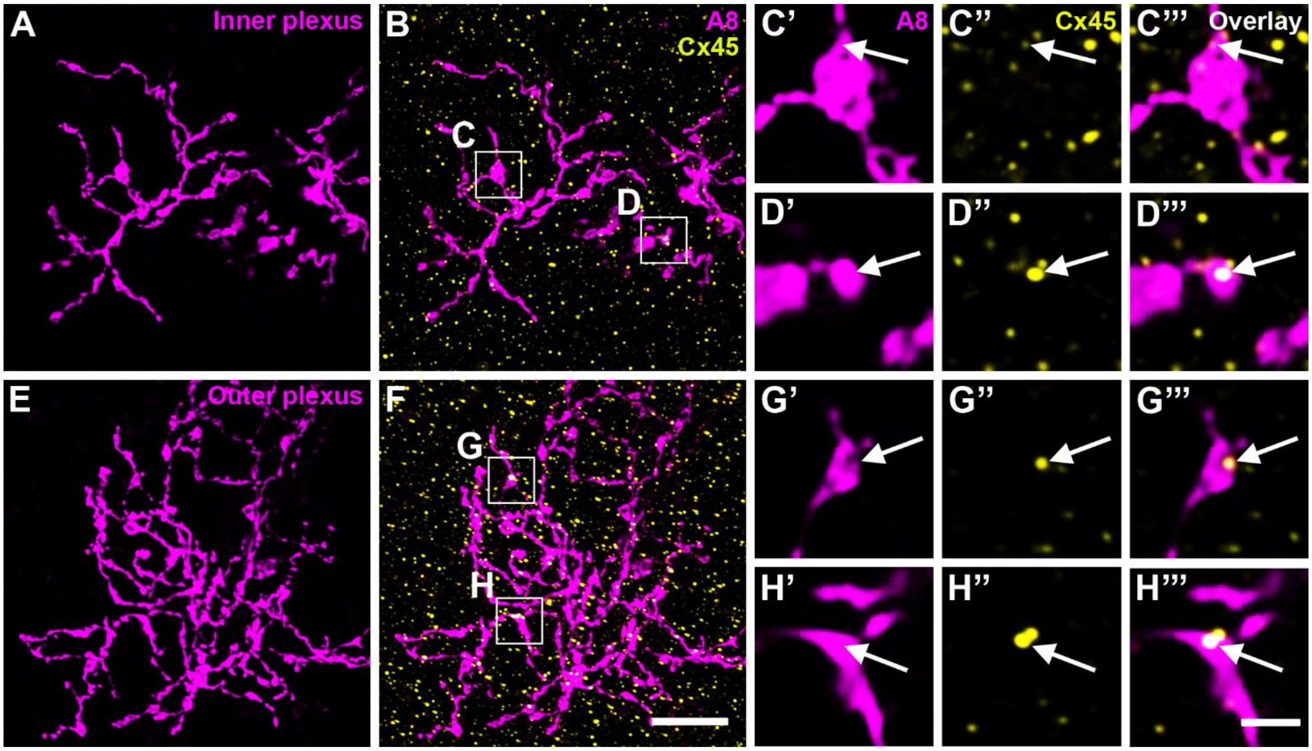

Figure S1. Gap junctions of A8 amacrine cells occasionally contain connexin45 (Cx45). (A, E) Maximum projection of inner **(A)** and outer dendrites **(E)** of an injected A8 cell. **(B, F)** Maximum

projections of the overlay of Cx45 with inner **(B)** and outer dendrites **(F)** of the injected cell. **(C'-D''', G-H''')** Selected areas from B and F as single, magnified sections: A8 dendrites **(C', D', G', H')**, Cx36 **(C'', D'', G'', H'')**, and the respective overlays **(C''', D''', G''', H''')**. Arrows point to colocalized Cx45-positive puncta on A8 cell dendrites. Scale bar: A, B, E, F, 10 µm; C'-D''', G'-H''', 2 µm.

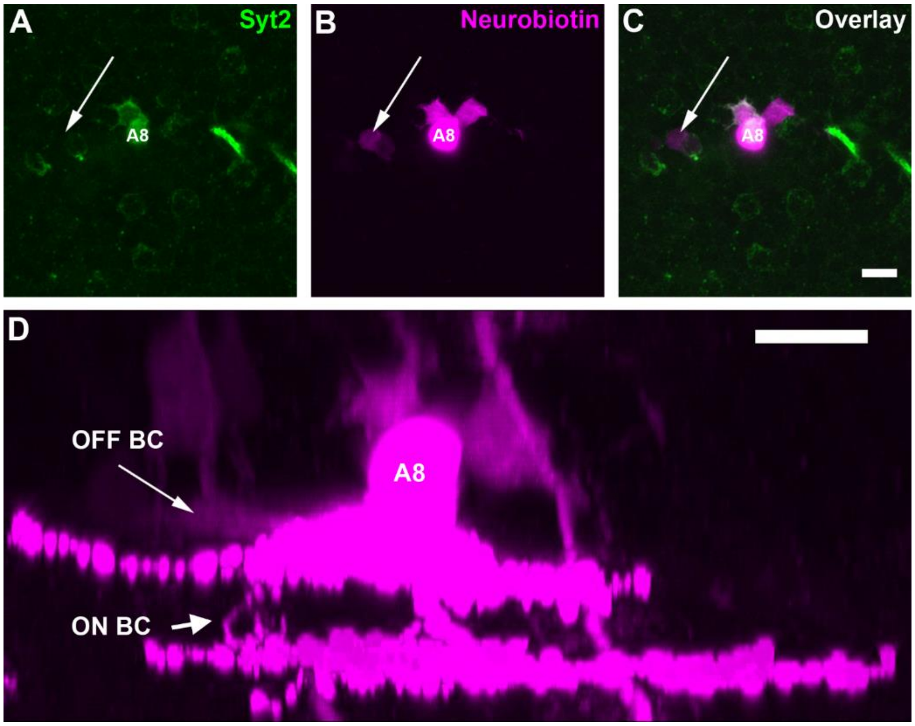

Figure S2. A8 cell tracer-coupled to a Syt2-negative OFF bipolar cell. (A) Syt2-negative bipolar cell (arrow) is coupled to a neurobiotin-injected A8 cell **(B)**. **(C)** shows the overlay of the maximum projections. A8 denotes the injected cell which also appears to express Syt2. However, this represents most likely bleed-through because the injected cell contains very high amounts of Alexa Fluor 546-conjugated streptavidin (to visualize the neurobiotin) and Alexa 568. **(D)** XZ rotation of the injected cell shown in **(A-C)**, revealing the dendrites of coupled OFF (arrow) and ON bipolar cells (short arrow). Please note that the dendrites of the injected A8 cell appear larger than they are because the cell was scanned with high gain to be able to visualize the dendrites of the coupled cells. Scale bar: A-C, 10 µm; D, 10 µm.

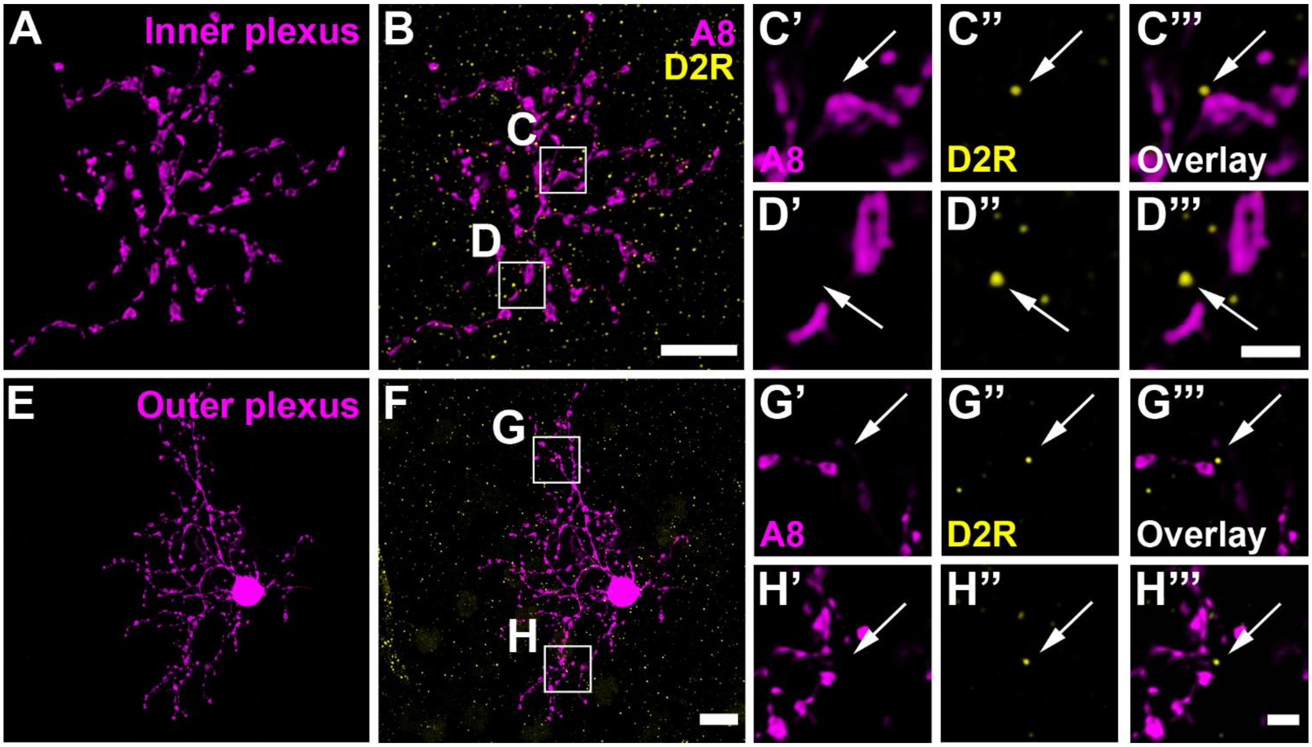

Figure S3. A8 cells did not express D_2 receptors. (A) Maximum projection of the inner dendrites of a dye-injected A8 cell. **(B)** Overlay of the A8 ON dendrites and D_2 receptors (D2R), shown as maximum projection. **(C, D)** Selected ROI from B. **(C'-D''')** Magnified images of A8 dendrites **(C', D')**, D_2 receptors **(C'', D'')**, and their respective overlay **(C''', D''')** within a single section from the selected ROI. Arrows point to D_2 receptors which did not colocalize with A8 ON dendrites. **(E-H''')** Same as **(A-D''')** for the outer plexus of an injected A8 cell, again revealing no colocalization between A8 dendrites and D_2 receptor immunoreactivity (arrows). Scale bar: A, B, 10 µm; E, F, 10 µm; C'-D''', G'-H''', 2 µm.

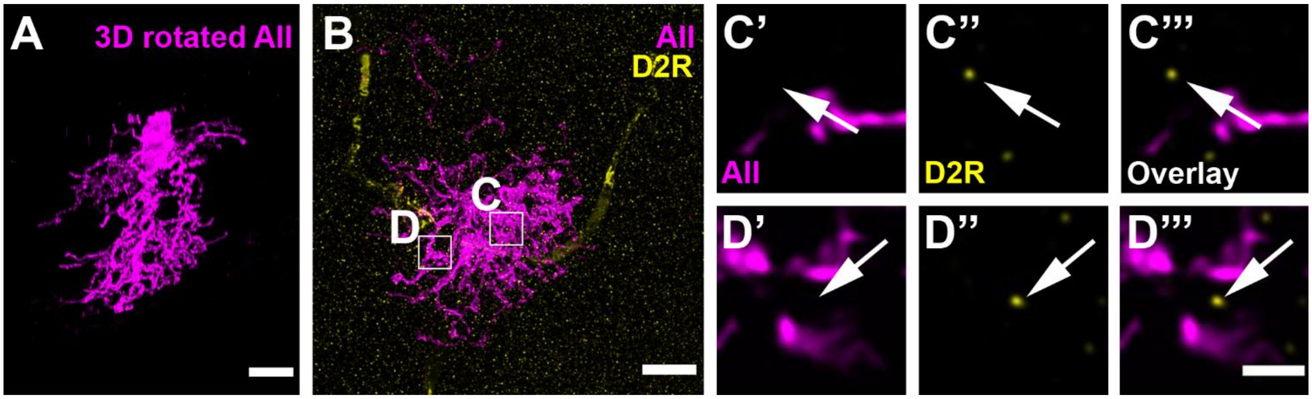

Figure S4. AII cells did not express D_2 receptors. (A) Maximum XZ rotation of an injected AII cell. **(B)** Maximum projection of AII cell dendrites in a retinal whole-mount labeled for D_2 dopamine receptors. Areas marked by the squares **(C, D)** are shown in higher magnification in C'-D'''. D_2 immunoreactivity (arrows) was never found on AII dendrites. Scale bar: A, B, 10 µm; C'-D''', 2 µm.

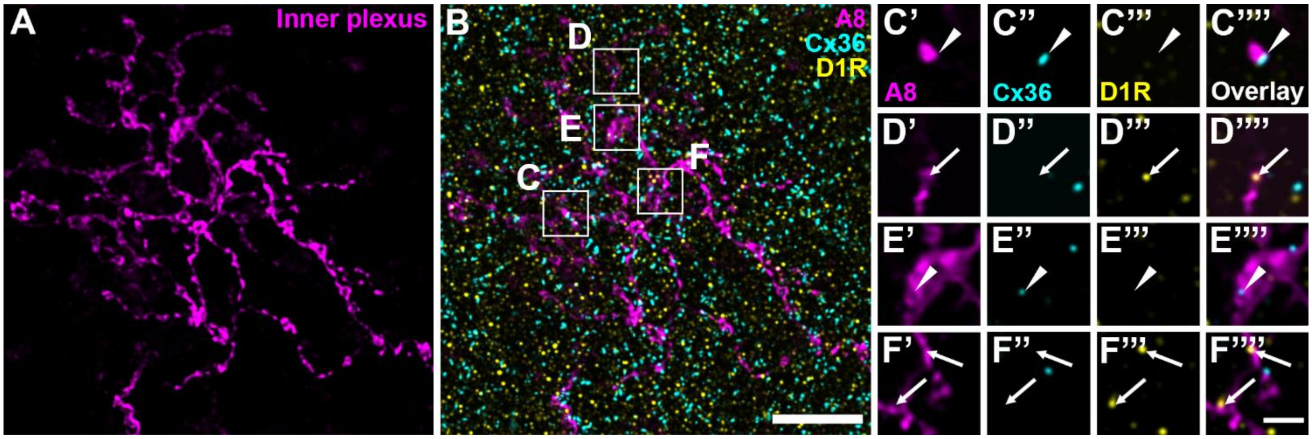

Figure S5. Immunoreactivities for Cx36 and D_1 receptors did not colocalize on A8 dendrites. (A) Maximum projection of the inner dendrites an injected A8 cell. **(B)** Maximum projections of the overlay of Cx36 and D_1 receptors and the inner plexus of A8 dendrites. Areas marked by the squares **(C-F)** are shown in higher magnification in C’-F’’’’. Cx36 is present on A8 dendrites (arrowheads, C’-C’’’’, E’-E’’’’) but not colocalized with D_1 receptors (arrows, D’-D’’’’, F’-F’’’’). Scale bar: A, B, 10 µm; C’-F’’’, 2 µm.

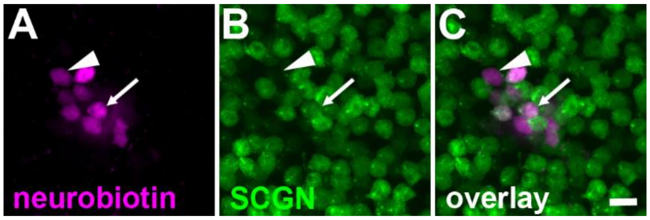

Figure S6. Secretagogin (SCGN)-positive and -negative bipolar cells were coupled to AII amacrine cells. (A) Maximum projection of AII-coupled bipolar cells. **(B)** Maximum projection of SCGN-positive bipolar cells. **(C)** Merged image of A and B. Arrows denote SCGN-positive bipolar cells coupled to AII amacrine cells. Arrowheads point to SCGN-negative bipolar cells. Scale bar: A-C, 10 µm.

2.2 Supplementary Tables

Table S1: Details for D2 receptor antibody used for Supplementary Figures S3 and S4.

Antibody	Host, type	Dilution	Source Cat., No.
D2 receptor	Mouse, monoclonal	1:250	UC Davis/NIH NeuroMab Facility, Davis, CA, 73-230

Table S2: Distribution of Cx36 and CtBP2 across the ON sublamina of the inner plexiform layer (IPL).

Number of puncta	ON IPL
Cx36	839 ± 338
CtBP2	2,190 ± 268
Colocalized	123 ± 28
Colocalized (%)	15.2 ± 3

Data obtained from two retinas, give as mean ± sd.

2.3 Supplementary Movie

Movie showing the inner plexus of an Alexa 568-injected A8 amacrine cell (magenta), immunolabeled for connexin36 (yellow). Please not that only a small fraction of all connexin36-positive puncta colocalize on the injected cell. Rotation is from -45 to 45 degrees.
